# From conceptualising to modelling structural determinants and interventions in HIV transmission dynamics models: a scoping review and methodological framework for evidence-based analyses

**DOI:** 10.1186/s12916-024-03580-z

**Published:** 2024-09-19

**Authors:** James Stannah, Jorge Luis Flores Anato, Michael Pickles, Joseph Larmarange, Kate M. Mitchell, Adelina Artenie, Kostyantyn Dumchev, Serge Niangoran, Lucy Platt, Fern Terris-Prestholt, Aditya Singh, Jack Stone, Peter Vickerman, Andrew Phillips, Leigh Johnson, Mathieu Maheu-Giroux, Marie-Claude Boily

**Affiliations:** 1 Department of Epidemiology and Biostatistics, School of Population and Global Health, McGill University, Montréal, Canada; 2 MRC Centre for Global Infectious Disease Analysis, School of Public Health, Imperial College London, London, UK; 3 HPTN Modelling Centre, Imperial College London, London, UK; 4 Centre Population et Développement, Institut de Recherche pour le Développement, Université Paris Cité, Inserm, Paris, France; 5 Department of Nursing and Community Health, Glasgow Caledonian University, London, UK; 6 Population Health Sciences, University of Bristol, Bristol, UK; 7 Ukrainian Institute On Public Health Policy, Kiev, Ukraine; 8 Programme PAC-CI, CHU de Treichville, Site ANRS, Abidjan, Côte d’Ivoire; 9 Department of Social and Environmental Health Research, London School of Hygiene and Tropical Medicine Faculty of Public Health and Policy, London, UK; 10 UNAIDS, Geneva, Switzerland; 11 The Johns Hopkins University School of Medicine, Delhi, India; 12 Institute for Global Health, University College London, London, UK; 13 Centre for Infectious Disease Epidemiology and Research, University of Cape Town, Cape Town, South Africa

**Keywords:** HIV, AIDS, Structural factors, Social determinants of health, Structural interventions, Mathematical modelling, Causal pathways, Mediation analysis, Conceptual framework, Key populations

## Abstract

**Background:**

Including structural determinants (e.g. criminalisation, stigma, inequitable gender norms) in dynamic HIV transmission models is important to help quantify their population-level impacts and guide implementation of effective interventions that reduce the burden of HIV and inequalities thereof. However, evidence-based modelling of structural determinants is challenging partly due to a limited understanding of their causal pathways and few empirical estimates of their effects on HIV acquisition and transmission.

**Methods:**

We conducted a scoping review of dynamic HIV transmission modelling studies that evaluated the impacts of structural determinants, published up to August 28, 2023, using Ovid Embase and Medline online databases. We appraised studies on how models represented exposure to structural determinants and causal pathways. Building on this, we developed a new methodological framework and recommendations to support the incorporation of structural determinants in transmission dynamics models and their analyses. We discuss the data and analyses that could strengthen the evidence used to inform these models.

**Results:**

We identified 17 HIV modelling studies that represented structural determinants and/or interventions, including incarceration of people who inject drugs (number of studies [*n*] = 5), violence against women (*n* = 3), HIV stigma (*n* = 1), and housing instability (*n* = 1), among others (*n* = 7). Most studies (*n* = 10) modelled exposures dynamically. Almost half (8/17 studies) represented multiple exposure histories (e.g. current, recent, non-recent exposure). Structural determinants were often assumed to influence HIV indirectly by influencing mediators such as contact patterns, condom use, and antiretroviral therapy use. However, causal pathways’ assumptions were sometimes simple, with few mediators explicitly represented in the model, and largely based on cross-sectional associations. Although most studies calibrated models using HIV epidemiological data, less than half (7/17) also fitted or cross-validated to data on the prevalence, frequency, or effects of exposure to structural determinants.

**Conclusions:**

Mathematical models can play a crucial role in elucidating the population-level impacts of structural determinants and interventions on HIV. We recommend the next generation of models reflect exposure to structural determinants dynamically and mechanistically, and reproduce the key causal pathways, based on longitudinal evidence of links between structural determinants, mediators, and HIV. This would improve the validity and usefulness of predictions of the impacts of structural determinants and interventions.

**Supplementary Information:**

The online version contains supplementary material available at 10.1186/s12916-024-03580-z.

## Background

Structural determinants of HIV are the social, economic, political, cultural, organisational, and environmental factors that shape HIV acquisition and transmission risks across individuals and populations (Table 1) [1–3]. Socio-ecological frameworks have been applied to understand how structural determinants influence HIV transmission dynamics among populations most vulnerable to HIV (i.e. key populations) [4, 5]. Key populations include people who inject drugs (PWID), men who have sex with men (MSM), transgender people, and female sex workers (FSW) [6]. Inequitable access to essential resources such as education, employment, and health care, coupled with the criminalisation of certain behaviours, including sex work, drug use, and same-sex relationships concentrates HIV vulnerabilities within these groups [4, 7–9]. This compounding effect is exacerbated by pervasive stigma, discrimination, racism, homophobia, and sexism [10].

**Table 1:** Definitions of key terms used in this paper

**Transmission dynamics model: **A model in which the force of infection changes over time due to direct and indirect effects from changes in the proportion of individuals living with transmissible HIV (i.e. virally unsuppressed) [26].
**Basic reproduction number,** *𝓡*_*0*_**:** The average number of secondary transmissions from a person living with HIV in an otherwise completely susceptible population. If *𝓡*_*0*_ >1, HIV has the potential to spread in the population, whilst if *𝓡*_*0*_ <1, sustained HIV transmission is unlikely. Conceptually, it depends on the contact rate (c), the duration of time virally unsuppressed (D), and the transmission probability per contact (β). Other factors also affect *𝓡*_*0*_, including population heterogeneity (vulnerability and exposure to HIV may vary across and within populations), and mixing patterns (how contact between groups varies, i.e. who mixes with whom) [27–30].
**Force of infection, *λ*:** The per capita incidence rate at which people susceptible in the population acquire infection [26]. It depends on the contact rate (c) (which can be conceptualised as accounting for mixing patterns by relevant population subgroups), the probability of transmission per effective contact (β), and the prevalence (I/N) of virally unsuppressed infection (I) among partners (N).
**Structural determinants:** The fundamental, foundational, underlying social, economic, political, cultural, organisational, and environmental determinants that affect HIV risks by shaping exposure patterns to risk and prevention factors (mediators) further downstream on the causal pathways [1].
**Distal structural determinants: **Macrolevel, aggregate structural determinants that affect whole populations, communities, or groups of individuals (e.g. key populations) [4, 31, 32]. They affect exposure to individual-level proximate structural determinants. Examples include laws and policies such as those governing sex work, sex between men, and drug use, but also alcohol and tobacco advertising, systemic and institutionalised racism, and inequitable norms surrounding gender, sexual identity, and substance use.
**Proximate structural determinants: **Structural factors experienced at an individual-level [4, 31, 32]. They are closer to and have more immediate effects on HIV risks. Examples include incarceration, stigma, discrimination, violence, housing instability, access, and availability of drugs.
**Structural interventions: **Interventions that promote the availability, accessibility, or acceptability of specific resources needed to prevent poor health outcomes or that reduce vulnerability to them [33]. They seek to mitigate the negative effects of structural determinants or prevent exposure to them (e.g. drug law reform that institutes drug treatment instead of incarceration). Structural interventions encompass both societal enablers and development synergies [34]. Societal enablers are social programmes, policies, and interventions that aim to remove barriers to accessing necessary health services. Examples include decriminalisation (e.g. of sex work, sex between men, and drug use/possession), community mobilisation, stigma reduction, and other specific interventions including the Avahan intimate partner violence intervention in India or the integration of self-help groups to empower FSW within the national sex worker programme in Zimbabwe [35, 36]. Development synergies are investments in other sectors that can have positive effects on HIV outcomes (e.g. HIV incidence, treatment use, mortality). Examples include investments in education, employment practices, gender equality, legal reform, as well as specific economic empowerment interventions, such as cash transfer interventions for women.
**Exposure history: **The specified duration of exposure as well as the time-varying intensities of exposure within different exposure periods (e.g. current, recent (< 6 months), and non-recent (≥ 6 months) exposures) [37]. Duration and time periods of exposure are usually based on the recall periods of the survey instruments that measure exposures, and intensities are based on the findings of analyses that assess the effects of exposure on causal pathways within those time periods.
**Causal pathways: **The chain of variables that causally link exposure to structural determinants and structural interventions to individual-level HIV risks.
**Direct pathways: **Causal pathways not involving mediators. These may represent that the mediators on the causal pathways are unmeasured and therefore unobserved.
**Indirect pathways: **Causal pathways that involve mediators.
**Mediators: **Intermediate variables on the causal pathways that link exposure to structural determinants and interventions to HIV risks. They are typically assumed or established as the main mechanisms through which exposure to structural determinants affects HIV vulnerabilities. Examples for structural determinants may include the number of sexual or injecting partners, the frequency of sex or sharing injection equipment, inconsistent condom use, and access to and uptake of HIV prevention and treatment, and others. Mediators may be observed or unobserved. The term ‘mediator’ to describe a variable is context specific. A variable that is a mediator on one causal pathway could be considered an independent exposure variable on another (e.g. pre-exposure prophylaxis (PrEP) use could be a mediator in analyses estimating the effect of exposure to HIV education on individual HIV acquisition risk and an exposure variable in analyses estimating the impact of PrEP use on HIV acquisition).
**HIV outcomes: **The last step in the causal pathways. These include HIV acquisition and onward HIV transmission, as well as individual-level HIV health outcomes such as HIV-related morbidity and mortality (e.g. disability-adjusted life years).

Recognising the importance of structural determinants, the *Global AIDS Strategy 2021–2026* includes the 10–10-10 targets [10]. These targets aim to reach < 10% of key populations and people living with HIV (PLHIV) experiencing stigma and discrimination, < 10% of women and key populations encountering gender-based inequalities and violence, and < 10% of countries having punitive laws and policies that limit access to HIV-related services by 2025. The global strategy commits to supporting community-led organisations to deliver 60% of HIV programmes on societal enablers (structural interventions that improve the effectiveness of HIV services) including those to reduce stigma and discrimination, support enabling legal environments, and eliminate gender-based violence [10]. However, quantitative evidence of the population-level contribution of structural determinants and the impact of structural interventions on HIV and other outcomes is sparse (although increasing), partly because these impacts are often difficult to evaluate empirically [11]. Estimating the population-level impact of structural determinants is required to inform effective policies and interventions to mitigate their impacts on HIV outcomes. It builds the evidence base on their importance and can inform resource allocation—through complementary economic evaluations—tailored to the most important epidemic drivers. Mathematical models of HIV transmission that carefully triangulate information on structural determinants can provide a means to estimate their population-level impacts and quantitatively account for uncertainty in their individual-level effects, even with sparse observed data, to generate evidence on the potential benefits of structural interventions [12]. A key benefit of these models is their ability to project non-linear dynamics, including both direct and indirect effects of structural determinants and interventions on HIV over relatively longer time horizons than statistical models when quantifying population-level impacts.

Transmission dynamic models that describe the acquisition and transmission of HIV have long been used to quantify the population-level impact of biomedical and behavioural interventions [13–16]. However, few mathematical models have so far considered structural determinants, in part due to the inherent complexity of incorporating these upstream factors, limited understanding of their causal pathways, and uncertainty in the benefits of associated interventions [11]. Unlike individual-level risk factors that directly influence HIV transmission, structural determinants influence HIV risks through multiple intervening mechanisms [2, 17]. Given the importance of structural determinants, a new generation of evidence-based mathematical models is needed to better inform public health and decision-making on ending HIV/AIDS, and to evaluate the cost-effectiveness of different intervention strategies. These models need to explicitly represent structural determinants in a way that adequately captures the patterns of exposure and their influence on individual-level HIV risks through different causal pathways, while being firmly grounded in robust empirical evidence.

The overarching objective of this paper is to develop an evidence-based methodological framework to improve the design and analysis of dynamic HIV transmission models of structural determinants. Using our experience of modelling structural determinants [4, 18–23] and a scoping review evaluating previous models that represented structural determinants of HIV, we develop recommendations for the next generation of models and data needs. Although our framework focuses on HIV, it can also be applied to other infectious diseases.

### Conceptual framework: causal pathways linking structural determinants to HIV in models

Structural determinants often have diffuse effects, in that exposure to structural determinants may impact multiple outcomes, through diverse causal pathways and mediators, which will differ by structural determinant and setting (see Table 1 for definitions of key terms) [17]. Exposure to some structural determinants may also increase exposure to other structural determinants (e.g. incarceration may increase exposure to stigma), and mediators and outcomes may themselves impact future exposure to structural determinants (e.g. HIV acquisition leading to illness, loss of income, and financial hardships) [24, 25]. Transmission dynamics models allow us to reproduce these complex relationships.

**Fig. 1:**
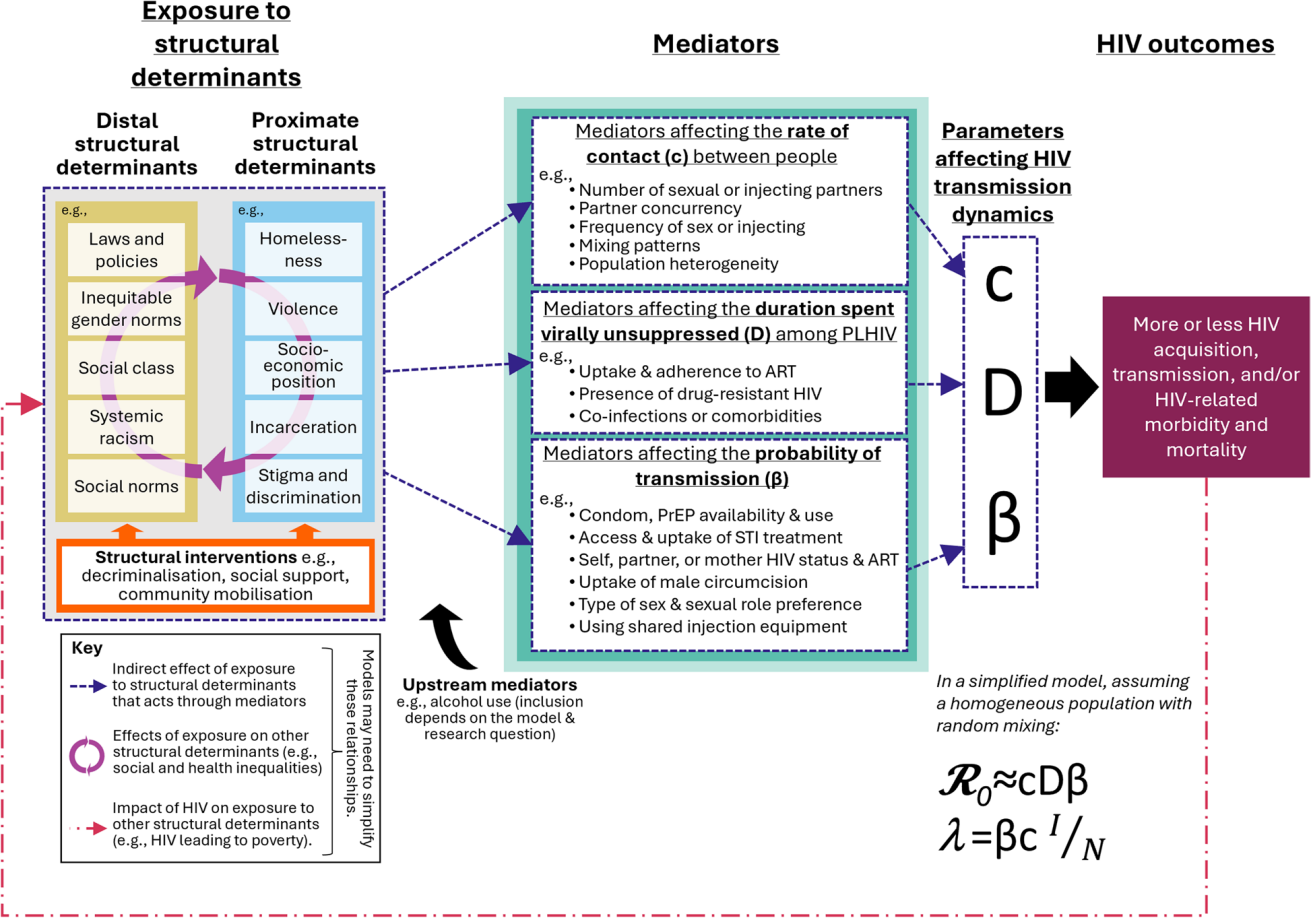
Conceptual framework illustrating the causal pathways connecting exposure to structural determinants to HIV transmission and population-level HIV outcomes, via mediators, in dynamic mathematical models. Exposure to distal structural determinants such as laws and policies and proximate structural determinants such as stigma and discrimination (e.g. homophobia, racism, sexism, transphobia) impact HIV outcomes through their effects on intermediate variables (mediators). How exposure to structural determinants may impact HIV transmission within a modelled population can be conceptualised by considering the effects of exposure to structural determinants and interventions on key parameters that determine the basic reproduction number, *𝓡*_*0*_, and the force of infection, *λ* (i.e. HIV incidence). In a simplified model that assumes a homogeneous population and therefore random mixing patterns, these parameters include contact rates (*c*), transmission probabilities (β), and the duration spent virally unsuppressed among PLHIV (*D*). Important mediators to account for include those affecting these parameters. In a more realistic heterogeneous population and models with non-random mixing, additional complexity can be considered. The exact way in which this is modelled will differ by model. ${}^{I}\!\left/ \!{}_{N}\right.$ = the prevalence of virally unsuppressed HIV among partners of those not living with HIV

To model exposure to structural determinants, we need to translate the main features of exposures into their mechanistic components. This requires identifying and defining the patterns of exposure to the structural determinants that can be modelled, based on available evidence of their prevalence and frequency in the populations and settings of interest. We then need to simulate the main causal pathways, including mediators, needed to adequately reproduce the effects of exposure on HIV outcomes (Table 1, Fig. 1). Ideally, this requires strong empirical evidence on the causal pathways, including mediators, and the magnitudes and durations of causal effects (e.g. relative risks) linking structural determinants, mediators, and HIV outcomes.

Structural determinants may be distal or proximate (Table 1, Fig. 1) [4, 31, 32, 38]. Distal structural determinants include macro-level aggregate exposures that affect whole populations, communities, or groups, such as laws and policies, social norms, and gender inequality [4, 31, 32]. Proximate structural determinants are individual-level consequences of distal exposures, such as incarceration, discrimination, and violence [4, 31, 32]. Some researchers advocate for focusing on proximate structural determinants, as they may be more easily modified by social programmes and policies [39]. They may also be more easily measured and thus operationalised in models, and their evidence base may be stronger than for distal structural determinants [5, 7, 40].

Models need to specify and quantify how exposure to structural determinants affects HIV outcomes, based on evidence of their effects. How these effects are captured in models will depend in large part on the model structure and the choice of mediators represented. In a simplified example modelling a homogeneous population with random mixing, important parameters that determine levels of HIV transmission include the probability of HIV transmission (β) per effective contact, the average duration of transmissibility among PLHIV (*D;* the time spent virally unsuppressed), and the contact rates between people (*c*; e.g. sexual or sharing injecting partners) (Fig. 1) [26]. Changes in these parameters influence the force of infection (*λ*) and the basic reproduction number (*𝓡*_*0*_)—concepts central to transmission dynamics models (Fig. 1, Table 1). In reality, populations are not homogeneous and both population heterogeneity and mixing patterns by relevant population subgroups will impact *𝓡*_*0*_ and *λ* and intersect with structural determinants [41, 42].

## Scoping review: existing models of structural determinants and HIV

### Search methods and studies identified

To develop our framework and recommendations, we conducted a scoping review of HIV transmission dynamic modelling studies to appraise previous approaches. We included studies that modelled exposures to structural determinants and/or interventions, and their mediators, and estimated their impacts on HIV acquisition and onward transmission in any population and setting. We conducted the search on August 28, 2023, for studies published since January 1, 1980, using Ovid Embase and MEDLINE online databases (Additional file 1: Text S1, Additional file 2: Tables S1 and S2). We adopted a three-way classification to characterise studies: (a) static approaches where the proportion of individuals exposed to the structural determinants and its effects on the assumed mediators and/or HIV acquisition or transmission risks were accounted for by applying fixed relative rates or probabilities to relevant model parameters influenced by the structural determinants; (b) stratification-based approaches where the modelled population could experience one level of exposure, with some movement between exposed and non-exposed states; and (c) stratification-based approaches with movement between multiple exposure history states (e.g. recent, non-recent). Our scoping review was reported using the PRISMA extension for scoping reviews (Additional file 2: Table S3) [43]. Additional details on the scoping review’s methods are provided in Additional file 1: Text S1.

We identified 17 modelling studies based on 13 models that assessed the impact of structural determinants and/or interventions on HIV (Table 2, Additional file 1: Text S2, Additional file 2: Table S4, Additional file 3: Fig. S1). Most studies modelled proximate structural determinants (number of studies [*n*] = 12) [4, 44–54] and/or structural interventions (*n* = 14) [4, 5, 44, 46–52, 54–57], primarily affecting key populations including PWID (*n* = 8) [5, 46–48, 50, 53–55], FSW (*n* = 5) [4, 49, 55–57], and MSM (*n* = 3) [5, 46, 55]. Four models of PWID were not gender-stratified [47, 50, 53, 54]. Studies were primarily published since 2015 (*n* = 13) [4, 44–48, 50–55, 58] and largely modelled settings in Western and Central Europe and North America (*n* = 8) [4, 5, 44–47, 49, 57] and Eastern and Southern Africa (*n* = 7) [4, 5, 49, 51, 52, 57, 58]. Seven studies modelled multiple settings in different regions [4, 5, 49, 53–55, 57], including two studies that modelled 58 and 77 countries, respectively [53, 55]. One study modelled hypothetical settings with moderate to high HIV prevalence [50].

The modelling objectives of studies were primarily to estimate the impact of structural interventions on new HIV acquisitions (*n* = 15) [4, 5, 44, 46–52, 54–58] or to assess the contribution of structural determinants to HIV epidemics (*n* = 6) [4, 45, 48, 51–53] (Table 2). Most studies estimated impacts by predicting the fraction of new HIV acquisitions occurring or averted under different scenarios (*n* = 11 [4, 5, 44, 45, 47, 49, 51, 54–57]; Table 2).

**Table 2:** Characteristics of HIV mathematical modelling studies identified in our scoping review

Reference	Type of model	Country	Population	Distal structural determinants	Proximate structural determinants	Structural interventions	Exposure histories represented	Additional exposure stratifications	Main mediators modelled	Main outcomes related to structural determinants/interventions
**a) Static approaches to representing exposure to structural determinants**										
Stover et al., 2021 [55]	Compartmental (Goals)	77 countries	Heterosexual men and women, FSW, MSM, and PWID	Criminalisation of drug use and sex work, inequitable norms and attitudes about PLHIV	Internalised HIV stigma among PLHIV, violence among women	**UNAIDS 10–10-10 (Decriminalisation of sex work and drug use, removing internalised HIV stigma, eliminating violence against women)**	Not applicable	Not applicable	Not represented	No. cumulative HIV acquisitions over 10 years (2020–2030) if UNAIDS 10–10-10 targets for 2025 are not achieved
Levy et al., 2021 [51]	Compartmental	Kenya	Heterosexual men and women	Inequitable norms and attitudes about PLHIV	**Internalised, enacted, and perceived HIV stigma**	**Stigma reduction**	Not applicable	Not applicable	ART use	No. annual HIV acquisitions over 13 years (2004–2017) compared to scenarios with different prevalence and rates of stigma
Ronoh et al., 2020 [58]	Compartmental	Kenya	Heterosexual men and women aged 15–24	**Positive and negative attitudes**^a^	**Positive and negative attitudes**^a^** affecting HIV testing, condom use, and ART use**	Not modelled	Not applicable	Not applicable	Condom use, HIV testing, ART use	Change in HIV prevalence over 5 years (2018–2023) comparing scenarios with different prevalence of positive and negative attitudes
Vassall et al., 2014 [56]	Compartmental	India	FSW	Criminalisation of sex work and policing practices, inequitable gender norms and attitudes towards sex workers	Stigma, discrimination, and violence against FSW	**Community mobilisation and empowerment for FSW**	Not applicable	Not applicable	Condom use	No. HIV acquisitions averted due to community mobilisation over the first 7 years of Avahan (2004–2011) comparing baseline to a scenario with no impact of community mobilisation on condom use
Wirtz et al., 2014 [57]	Compartmental (Goals)	Kenya, Thailand, Brazil, Ukraine	Heterosexual men and women, including FSW	Criminalisation/regulation of sex work, lack of safe spaces for sex work, inequitable gender norms and economic opportunities for women, attitudes towards sex workers	Stigma and discrimination against FSW	**Community empowerment for FSW**	Not applicable	Not applicable	Condom use, ART effectiveness	No. HIV acquisitions averted over 5 years (2011–2016) comparing scenarios with varied intervention coverage
Decker et al., 2013 [49]	Compartmental (Goals)	Ukraine, Kenya	FSW and non-FSW (gender-stratified)	Criminalisation/regulation of sex work, inequitable gender norms and attitudes towards sex workers	**Violence against FSW**	**Reducing violence against FSW**	Not applicable	Not applicable	Condom use	Cumulative HIV acquisitions averted over 5 years (2011–2016) comparing to scenarios with reduced prevalence of violence
Strathdee et al., 2010 [5]	Compartmental	Ukraine	PWID, including heterosexual men and women, bisexual MSM, and exclusive MSM	Criminalisation of drug use and policing practices	Police beatings among PWID (Ukraine)	**Elimination of police beatings in Ukraine and scale-up of opioid agonist therapy, needle and syringe programmes, and ART**	Not applicable	Opioid agonist therapy and needle and syringe programme status	Sharing injection equipment	Percentage of HIV acquisitions averted over 5 years (2010–2015) comparing baseline in each setting to scenarios with no police beatings
**b) Stratification-based approaches to representing structural determinants, where the modelled population could experience one level of exposure, with some movement between exposed and non-exposed states**										
Rigby and Johnson, 2017 [52]	Individual-based	South Africa	Heterosexual men and women	Inequitable gender norms	**Intimate partner violence against women**	**Violence reduction based on two interventions (IMAGE and SASA!)**	No IPV in partnership, IPV in partnership. Once there is IPV, partnerships remain violent for their duration	Relationship length, sexual risk behaviour group, predisposition and susceptibility to violence	Condom use, relationship dissolution, marriage rate, number of secondary partners, viral suppression, mixing patterns	HIV PAF of violence over 25 years (1990–2015) comparing baseline to a scenario with no IPV. Reduction in HIV incidence over 10 years (2015–2025) due to both interventions
Stone et al., 2022 [53]	Compartmental	58 countries	PWID (not gender-stratified)	Criminalisation of drug use, inequitable norms and attitudes about PWID, economic inequality	**Housing instability among PWID**	Not modelled	Not unstably housed, unstably housed	None	Mediators not represented, but total effect of exposure on HIV assumed to capture pathways involving injecting drug use	Global and country-level tPAFs of unstable housing among PWID over 10 years (2020–2030) by comparing baseline for each setting to scenarios with no impact of unstable housing on HIV
**c) Stratification-based approaches to representing structural determinants with multiple exposure histories**										
Shannon et al., 2015 [4]	Compartmental	Canada, India, Kenya	FSW and clients	Criminalisation of sex work, inequitable gender norms and attitudes towards sex workers, and safety of sex work environment	**Violence against FSW**	**Various hypothetical interventions including elimination of sexual violence, decriminalisation of sex work, increasing safer sex work environments, community empowerment and outreach**	Never, recent (< 6 or 12 months), and non-recent (> 6 or 12 months) client physical violence, client sexual violence, or police harassment. Type of violence and exposure history were setting-specific	Work environment, PWID status (Canada), member of sex worker collective (India), binge drinking (Kenya)	Condom use	Percentage of cumulative HIV acquisitions averted over 7 years (2014–2021) comparing baseline in each setting to various scenarios (e.g. setting violence rates to zero to simulate eliminating violence, removing the excess risk due to lower condom use among FSW ever exposed to simulate counselling)
Ward et al., 2022 [54]	Compartmental	Belarus, Russia, Kazakhstan, Kyrgyzstan	PWID (not gender-stratified)	Criminalisation of drug use	**Incarceration of PWID**	**Drug law reform**	Never, currently, recently (< 6 months) and non-recently incarcerated (> 6 months)	PWID status, opioid agonist therapy status	Mediators not represented, but total effect of exposure on HIV assumed to capture change in frequency of sharing injection equipment, mixing patterns	Percentage of HIV acquisitions averted over 20 years (2020–2040) comparing baseline to different scenarios (e.g. setting incarceration rates to zero to simulate decriminalisation, and opioid agonist therapy and ART scale-up)
Adams et al., 2021 [44]	Individual-based (TITAN model)	USA	African American men and women. Only men can be incarcerated	Racial biases in arrests and sentencing, inequitable gender norms	**Incarceration of African American men**	**Different PrEP prescription strategies for women with incarcerated male partners**	Never, currently, recently (< 6 months) and non-recently incarcerated (> 6 months). Higher incarceration rates if previously incarcerated	Type of incarceration facility	Relationship dissolution, number of sexual partners, probability of current STI, ART use, mixing patterns	No. of cumulative HIV acquisitions averted over 10 years (2015–2025) comparing baseline to different scenarios of PrEP scale-up among female partners of incarcerated men, and incarceration rates
Bernard et al., 2020 [46]	Individual-based	USA	PWID, people who use drugs, MSM, and lower-risk heterosexuals (gender-stratified)	Criminalisation of drug use and possession	**Incarceration of PWID**	**Jail diversion programme for low-level drug offenders**	Not incarcerated, currently in jail or prison, currently in drug court, currently in diversion program	Type of crime, jail further stratified by whether awaiting court or serving sentence	Mixing patterns, use of needle and syringe programmes, substance use disorder treatment, and ART, frequency of sharing, mixing patterns	Reduction in HIV incidence over 10 years (years not specified) by comparing baseline to a scenario with no jail diversion
Adams et al., 2018 [45]	Individual-based (TITAN model)	USA	African American men and women. Only men can be incarcerated	Racial biases in arrests and sentencing, inequitable gender norms	**Incarceration of African American men**	Not modelled	Never, currently, recently (< 6 months) and non-recently incarcerated (> 6 months). Higher incarceration rates if previously incarcerated	Type of incarceration facility	Relationship dissolution, number of sexual partners, probability of current STI, ART use, mixing patterns	No. cumulative HIV acquisitions averted among women over 10 years (2005–2015) comparing baseline to a scenario with no incarceration
Borquez et al., 2018 [48]	Compartmental	Mexico	PWID (gender-stratified)	Criminalisation of drug use and possession	**Incarceration of PWID and syringe confiscation by police**	**Drug law reform that institutes drug treatment instead of incarceration, compulsory abstinence programme**	Incarceration: Never, current, recently (< 6 months), non-recently incarcerated (> 6 months) Syringe confiscation: confiscation (< 6 months), no confiscation	Opioid agonist therapy status, rehabilitation (compulsory abstinence programme) status	Frequency of sharing, opioid agonist therapy use, mixing patterns	HIV PAF of incarceration and syringe confiscation over 18 years (2012–2030) and 5 years (2012–2017) comparing baseline to scenarios with no incarceration or impacts of recent incarceration and syringe confiscation to simulate full drug reform
Altice et al., 2016 [47]	Compartmental	Ukraine	PWID (not gender-stratified)	Criminalisation of drug use and possession	**Incarceration of PWID**	**Stopping incarceration of PWID and scale-up of prison-based opioid agonist therapies**	Never, currently, recently (< 12 months), non-recently incarcerated (> 12 months)	Opioid agonist therapy status	Mixing patterns. Other mediators not represented, but total effect of exposure on HIV assumed to capture change in frequency of sharing injection equipment	Percentage of HIV acquisitions averted and PAF of incarceration over 15 years (2015–2030) comparing baseline to different scenarios (e.g. setting incarceration rates to zero, scaling up opioid agonist therapy, removing the excess risk if recently incarcerated)
Dolan et al., 2016 [50]	Compartmental	Hypothetical moderate and high-prevalence settings	PWID and non-PWID (not gender-stratified)	Criminalisation of drug use and possession	**Incarceration of PWID**	**Reduced incarceration, scale-up of prison-based and post-release opioid agonist therapy, retention on ART post-release**	Never, current, recently (< 6 months), non-recently incarcerated (> 6 months)	PWID status	Frequency of sharing injection equipment, mixing patterns	Percentage reduction in HIV incidence over 5 years (years not specified) and reduction in incarcerated PWID comparing baseline to scenarios with reduced incarceration rates

Bolded structural determinants and interventions are those that were represented in models. For each, we also noted the distal and/or proximate structural determinants linked to the primary structural determinants and interventions modelled, which were not explicitly represented in any model

*ART* anti-retroviral therapy, *FSW* female sex workers, *IMAGE* Intervention with Microfinance for AIDS and Gender Equity, *MSM* men who have sex with men, *PAF* population attributable fraction, *tPAF* transmission population attributable fraction, *PWID *people who inject drugs, *STI *sexually transmitted infection, *TITAN *Treatment of Infection and Transmission in Agent-based Networks model

^a^Positive attitudes represent e.g. confidentiality by health workers, adequate support structure at home and community, and improved financial status. Negative attitudes represent e.g. religion, peer influence, perceived risk, stigma, poverty, caregivers’ waning support, confidentiality breaches by health workers and others

### Structural determinants and interventions examined

Exposure to proximate structural determinants included incarceration of PWID (*n* = 5) [46–48, 50, 54] and African American men (*n* = 2) [44, 45], client- and police-perpetrated violence against FSW (*n* = 2) [4, 49], intimate partner violence against women (*n* = 1) [52], HIV stigma (*n* = 1) [51], and housing instability among PWID (*n* = 1) [53]. Few studies modelled distal exposures (Table 2, Additional file 2: Table S4) [58]. One study modelled “positive and negative attitudes” among Kenyan youth, which reflected a combination of proximate and distal exposures (e.g. health worker confidentiality, poverty, peer influences, stigma, and more) [58]. The modelled structural interventions included reducing/eliminating incarceration of PWID (*n* = 5) [46–48, 50, 54], reducing/eliminating violence against women and FSW (*n* = 5) [4, 5, 49, 52, 55], community mobilisation and empowerment for FSW (*n* = 3) [4, 56, 57], and HIV stigma reduction [51]. Most of these modelled several interventions or delivery strategies. One study considered the impacts of achieving the UNAIDS 10–10-10 targets [55]. Another modelled structural changes, including eliminating police beatings in Ukraine [5]. Six studies, five of which modelled incarceration, also modelled scale-up of biomedical interventions such as prison- or community-based opioid agonist therapy, PrEP, or ART for prisoners or their partners [5, 44, 47, 48, 50, 54].

### Representations of exposure to structural determinants

The static representation category included studies that did not explicitly represent structural determinants (e.g. as compartments; *n* = 7; Table 2 (a), Additional file 2: Table S4) [5, 49, 51, 55–58]. For instance, Strathdee and colleagues modelled the impact of eliminating police beatings among PWID in Ukraine by comparing the baseline to a scenario with reduced sharing of injection equipment by a factor that was informed by empirical analyses showing greater sharing frequency if ever beaten by police and assuming that the reduction in sharing was due to the elimination of beatings [5]. Studies in this category included others that represented structural determinants as parameters that influenced HIV transmission or behaviours (*n* = 3) [5, 51, 58], and studies using the Goals models (*n* = 3) [49, 55, 57].

**Fig. 2:**
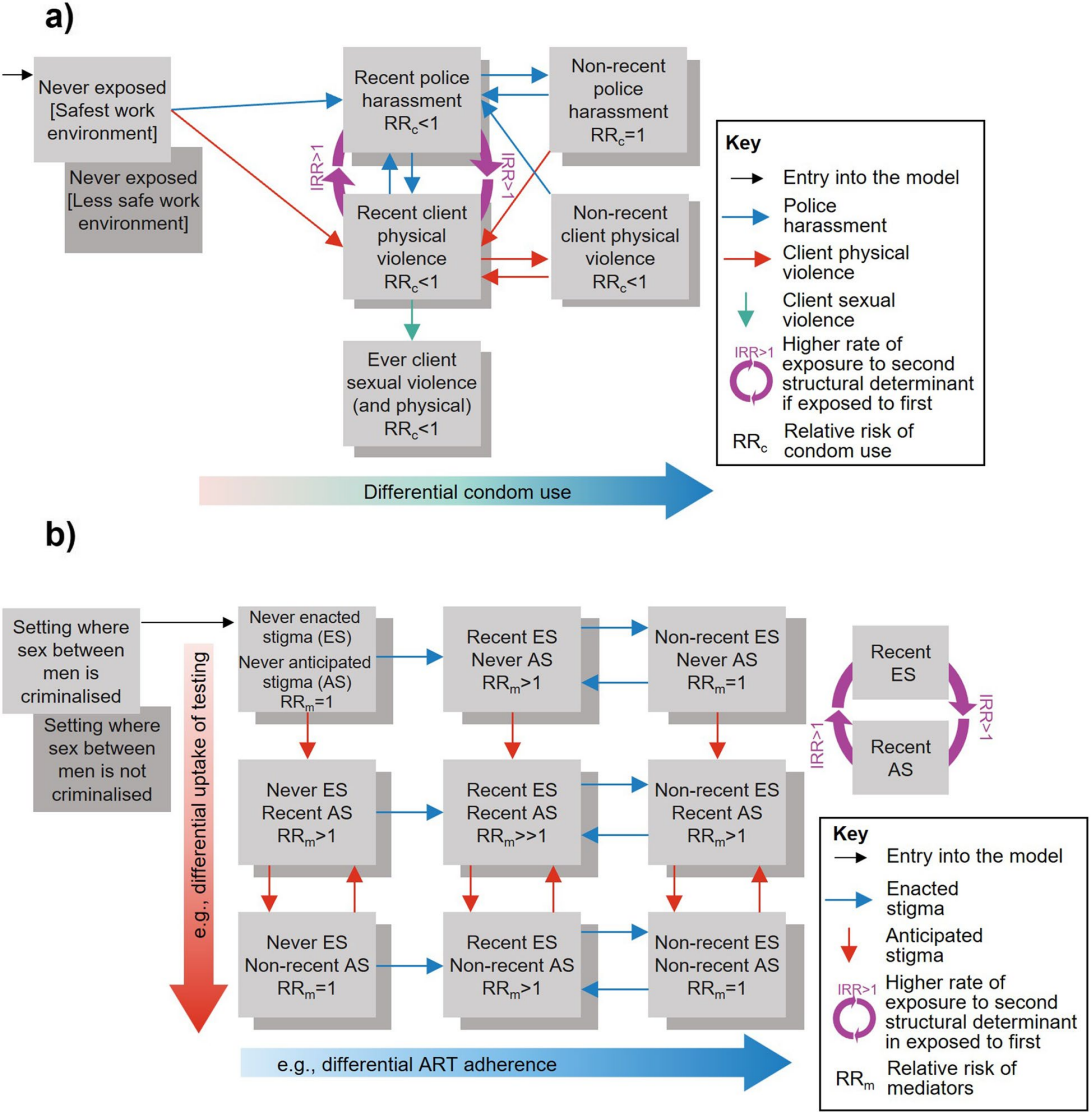
Dynamically representing exposure to structural determinants and their causal pathways in HIV models, with multiple different exposures and exposure histories. **a** Model flowchart (adapted from Shannon et al., 2015) [4] showing how exposure to different types of violence among FSW in different work environments and their impacts on HIV were represented in their model, and **b** a hypothetical model flowchart based on Shannon’s approach representing how exposure to stigma among MSM in settings could be modelled. Evidence suggests that in settings where sex between men is criminalised, MSM experience more stigma [59]. Enacted stigma, such as denial of care, and anticipated stigma, such as fear of discrimination, are linked to lower and slower uptake of HIV testing and treatment [60]. These could be represented by stratifying the population based on type of stigma, and criminalisation of sex between men, with multiple exposure histories for stigma to reflect short and long-term effects of exposure on HIV risks, and interactions reflecting links between the different exposures (purple arrow, incidence rate ratio for exposure; IRR > 1)

The stratification-based representation category included studies that stratified the population into mutually exclusive compartments or states, with transitions between them, to represent one current or recent exposure history to structural determinants (*n* = 2; Table 2 (b), Additional file 2: Table S4) [52, 53] or that represented multiple different exposure histories (*n* = 8; Table 2 (c), Additional file 2: Table S4) [4, 44–48, 50, 54]. For example, Shannon and colleagues’ model among FSW in Canada, Kenya, and India was the first to represent several structural determinants and exposure histories dynamically (Fig. 2a provides a simplified adaption of their Vancouver model flowchart) [4]. FSW transitioned between compartments of never, recent, and non-recent physical and sexual client violence and police harassment, which differed by settings. Similarly, all studies of incarceration represented multiple exposure histories (e.g. current, recent, non-recent incarceration; Table 2, Additional file 2: Table S4) [44, 45, 47, 48, 50, 54].

Dynamically representing structural determinants offers more flexibility to capture both long and short-term effects of exposure, including cumulative, gradual, waning, or lagged effects, by varying HIV risks associated with each exposure level. It also allows for the consideration of different rates of re-exposure. Granular exposure histories also facilitate a wider range of interventions to be explored. For instance, Shannon’s model differentiated the smaller impact of an intervention that reduces the incidence of violence versus an intervention that additionally removes the persisting negative effects of ever having experienced violence [4]. It showed that tackling all forms of violence would have a greater impact on HIV given high levels of co-exposures and interactions. Other structural determinants could be modelled similarly (Fig. 2b provides an example for stigma among MSM). Nevertheless, the stratification-based approach can be complex and data-intensive, making the static approach perhaps more practical for situations with sparse data, such as initial assessments. However, the stratification-based approach can also be simplified by using fewer stratifications.

Most studies represented the indirect effects of exposure to structural determinants through mediators related to sexual behaviours and HIV services access (Table 2). For instance, Shannon’s study modelled the effects of exposure to violence on HIV through lower condom use, and feedback loops between the types of violence, since recent police harassment increased exposure to recent client violence and vice versa (Fig. 2a) [4]. The most common mediators across studies were contact patterns (*n* = 8) [44–48, 50, 52, 54], the frequency or number of sexual/injecting partners (*n* = 9) [5, 44–48, 50, 52, 54], condom use (*n* = 6) [4, 49, 52, 56–58], and ART use (*n* = 5) [44–46, 51, 58]. Some studies considered upstream mediators, such as binge drinking or harm reduction services [4, 46–48]. Three studies among PWID (two on incarceration [47, 54] and one on housing instability [53]), modelled both the total effect of exposure (by changing the transmission probability among those exposed based on empirical estimates that implicitly captured indirect pathways involving injection drug use) and indirect effects through changes in mixing patterns (e.g. no contact between those in prison and the community; Table 2).

### Use of empirical evidence

In all studies, empirical evidence was used to inform model development. The information used included the proportion of the population exposed to the structural determinants (*n* = 9) [44–46, 48–52, 54], rates of exposure (*n* = 5) [4, 44, 45, 47, 48, 50, 52, 54], durations of exposure (*n* = 7) [44–47, 50, 53, 54], and estimates of the effect size of exposure to structural determinants or interventions on mediators or HIV risks (*n* = 10) [4, 5, 44, 45, 48, 50, 52–57] (Additional file 2: Table S5). All models were calibrated to different HIV outcomes (e.g. HIV prevalence, ART coverage) (Additional file 2: Table S6). Seven studies also calibrated or cross-validated models using structural determinants data including the proportion exposed or exposure rates (*n* = 4) [47, 51–53], and the effect size of exposure on HIV or HIV prevalence or incidence stratified by exposure histories (*n* = 4) [47, 48, 50, 54] (Additional file 2: Table S6).

Most model assumptions on structural determinants and their effects on mediators and HIV risks were based on empirical evidence—mostly from surveillance data or cross-sectional surveys, and largely from the same settings and risk populations as those modelled (Additional file 2: Table S5). In these modelling studies, the effects of exposures on mediators and HIV risks were based on various designs, each with limitations, including cross-sectional studies, cohort studies, trials, and some systematic reviews and meta-analyses, although these mostly included cross-sectional studies and sometimes pooled data from multiple settings. Cross-sectional effect sizes may limit the strength of evidence of a causal link, due to reverse causation. Single parameters were often informed by multiple sources (Additional file 2: Table S5). Only one study (Shannon et al.) that represented structural determinants dynamically with multiple different exposure histories was informed by longitudinal data on the effects of exposure on its mediators (condom use) for all exposure histories, and only in one of the three settings modelled [4]. Data used to parameterise exposures, transitions, and effect sizes were sometimes derived from different studies and settings, meaning that estimates informing the same model were not always based on standardised exposure definitions, potentially reducing the external validity of some model findings. Few studies validated their model predictions for structural determinants against observed estimates, perhaps due to insufficient validation data [4].

## Methodological framework: improving models of structural determinants and HIV

**Fig. 3:**
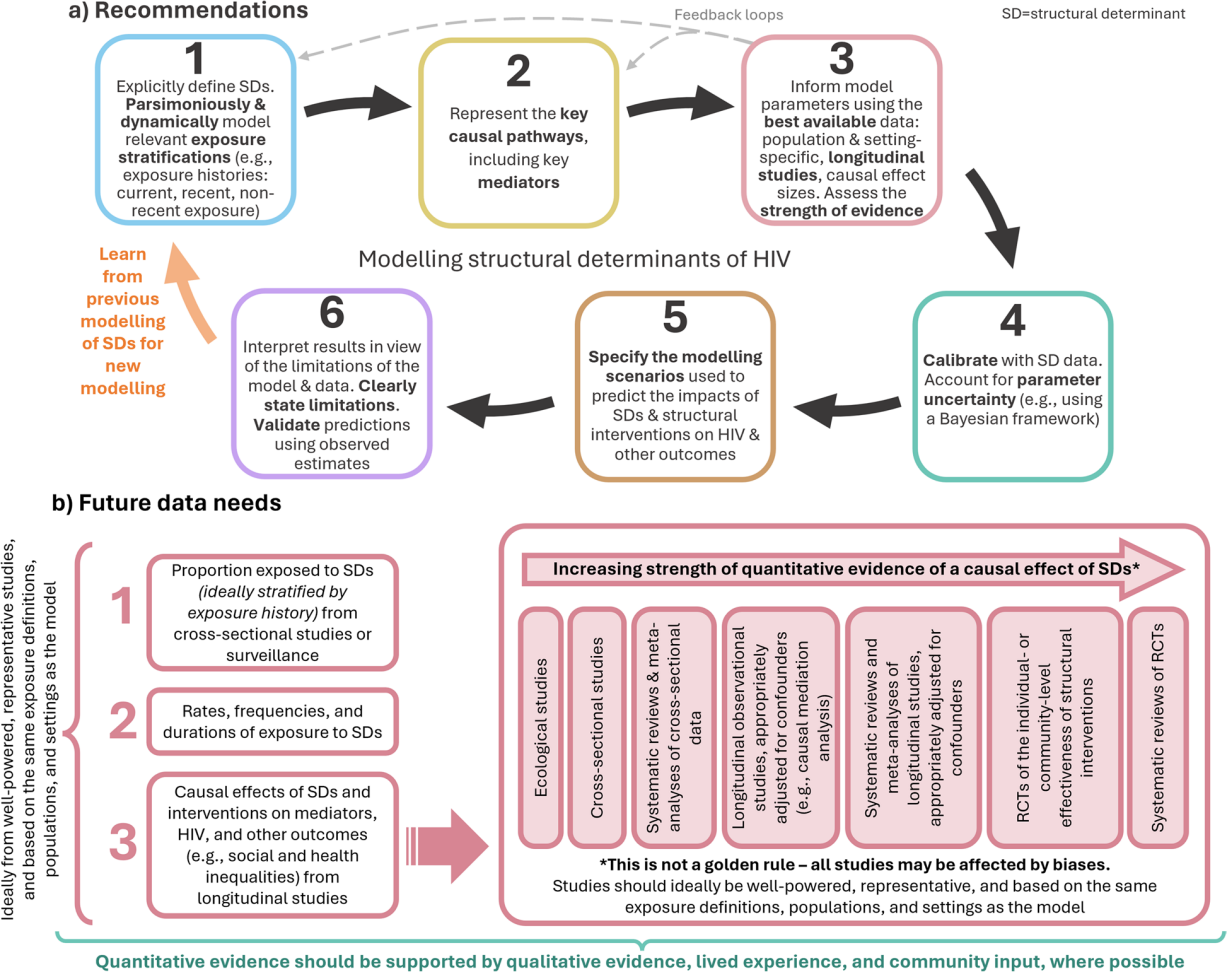
Methodological framework for modelling structural determinants. **a** Recommendations for the next generation of models focused on structural determinants and HIV, and **b** the future data needed to improve models of structural determinants, including the strength of quantitative evidence that could be used to inform the effects of exposures on mediators and HIV outcomes in models. SD, structural determinant

Given existing limitations, we propose a generalised framework of recommendations for modelling structural exposures and their causal pathways and discuss data needs for this next generation of models (Fig. 3, Table 3). For simplicity, we focus on deterministic compartmental models, but the framework can also be applied to individual-based models.

**Table 3:** Recommendations for developing, analysing, and describing models of exposure to structural determinants and interventions

Topic	No	Recommendation to consider
**1. General**		
*** Structural determinants***	1.1	Clearly define the structural determinant(s) of interest
*** Population, setting, and time period***	1.2	Specify the population group(s) exposed to the structural determinant, the setting(s) modelled, and the year(s) modelled
*** Research objectives & research questions***	1.3	Define i) the objectives of the modelling exercise (e.g. predicting the contribution of exposure to past epidemics and/or the impact of structural interventions on new transmissions) and ii) the research questions
**2. Exposure to structural determinants**		
*** Exposure history***	2.1	Consider reflecting different exposure histories (e.g. current, recent, and non-recent exposure) to account for short- and long-term exposure effects
*** Additional stratifications***	2.1a	Consider additional stratifications (e.g. different durations, frequencies, intensities, exposure environments, etc.) that could be needed to replicate the effects of exposure in the model
*** Influence of past exposures***	2.2	If relevant to the structural determinant, consider reflecting the influence of past exposures on future risks of exposure (e.g. reincarceration rates)
*** Co-exposures and inter-relationships***	2.3	If modelling multiple structural determinants, consider representing interrelationships between them (i.e. interactions)
*** Influence of interventions***	2.4	If modelling structural interventions, describe the interventions and explain how they are assumed to influence exposure to structural determinants (as defined above) or causal pathways (as described below)
**3. Causal pathways and mediators**		
*** Causal pa****t****hway overview***	3.1	Represent the modelled direct and indirect causal pathways from exposure to mediators, and HIV risks in flowcharts
***Mediators***	3.1a	Clearly define the mediators on indirect pathways
*** Effect size estimates***	3.2	Specify the magnitude of direct and indirect effects of structural exposures on mediators and/or HIV risks
*** Intervention pathways***	3.3	If modelling structural interventions, describe how they impact the causal pathways they intervene on
**4. Empirical evidence, model parameterisation, and calibration**		
*** Evidence-based***	4.1	Ensure that causal pathways and mechanisms of interventions are evidence-based
*** Parameterisation***	4.2	Parameterise the model using data (point estimates and uncertainty ranges) on exposures, mediators, and their effects on HIV prevalence and/or incidence, preferably from the same settings and populations as those modelled
*** Calibration***	4.3	Calibrate the model using epidemiological as well as structural determinants data (point estimates and 95% confidence/uncertainty intervals), accounting for parameter uncertainty (e.g. using a Bayesian framework)
*** Qualitative and other sources of evidence***	4.4	Consider whether model assumptions and causal pathways are also supported by qualitative evidence, social theory, and/or input from people with lived experiences
**5. Model outcomes, modelling scenarios, and validation**		
*** Main model outcomes***	5.1	Define the primary model outcomes (e.g. infections averted, PAF, tPAF, HIV prevalence or incidence) and secondary outcomes (e.g. impacts on other structural determinants or the mediators) and provide uncertainty ranges of model estimates
*** Time horizons of outcomes***	5.2	Determine the time horizon of analyses. Consider predicting outcomes over short (1 year), medium (2–10 years), and longer (> 10 years, lifetime, etc.) time horizons to understand short, medium, and long-term impacts of exposures and interventions
*** Modelling scenarios***	5.3	Specify the modelling scenarios, including counterfactuals, used to estimate model outcomes and address the primary (and secondary) research questions
*** Sensitivity analyses***	5.4	Use sensitivity analyses to explore how impacts change if short-term reductions in exposure are not sustained long-term
*** Validation***	5.5	Validate model estimates of the proportion exposed and individual- and population-level impacts of exposures, interventions, and mediators by comparing to empirical estimates that were not used for fitting

*PAF* population attributable fraction, *tPAF* transmission population attributable fraction

### Recommendations

First, models should consider dynamic and granular representations of structural determinants within the model, while being cautious not to add complexity when there is not strong evidence to support it (Fig. 3a). Models should represent the key dimensions of exposure, including exposure histories, duration, frequency, intensity, as well as co-exposures with other structural determinants and important feedback loops linking them. To connect exposure to HIV outcomes, the key causal pathways should be considered, including the mediators required to adequately capture the effects of exposure in the model.

When deciding on parameters related to structural determinants, it is important to weigh up the strengths and validity of available evidence and their relevance to the specific research question and context. Even if the model perfectly represents the mechanistic process linking structural determinants to HIV outcomes, using biased inputs, or inputs from different populations and settings, could bias model outputs [61]. Ideally, modellers should consider evidence for effect modification, cumulative effects, and interactions [62]. If parameters are uncertain and the internal validity is weak, transparently conducting detailed uncertainty and sensitivity analyses is warranted [63]. In some instances, modellers may need to decide whether to try and incorporate uncertainty in the appropriate parameter value, explore assumptions in additional scenarios, or not to model the research question at all. Attention should be paid to the external validity (i.e. generalisability and transportability) of parameters [64]. At the fitting stage, data on HIV epidemiological and intervention outcomes should be used, ideally stratified by exposure history to the structural determinant. Efforts should be made to fit or validate model predictions to the prevalence of exposure to structural determinants, and levels of mediators by exposure history, if available and relevant. Ideally, the fitting method should allow uncertainty in parameter assumptions to be reflected (e.g. using a Bayesian framework), including uncertainty in estimates related to structural determinants [26].

Finally, when conducting model analyses, the modelling scenarios, including the counterfactuals, used to assess the contribution of structural determinants to HIV incidence or to evaluate future changes due to introducing structural interventions should be clearly specified. Sensitivity analyses should be used to explore how impacts change if short-term reductions in exposure to structural determinants are not sustained long-term [63]. Data on HIV outcomes, mediators, and structural determinants not used at the fitting stage should be used to validate predictions, which can help indicate whether the model predicts the impact of structural interventions well or not [65]. Similarly, predictions from older models considering the same structural determinants could be compared to observed estimates, to identify strengths and weaknesses in their model structures and/or parameterisations that can inform newer models.

### Future data needs

Ultimately, the extent of model complexity will be determined by the research question and the availability of data on structural determinants, mediators, confounders, and HIV or other outcomes (Fig. 3b). Our set of recommendations (Table 3, Fig. 3a) can help outline data issues to consider. Ideally, exposures to specific structural determinants would be consistently measured to facilitate comparisons across studies and from the same settings and populations modelled. However, currently, exposure measurements (i.e. the survey questions) can vary considerably. For example, a global systematic review among sex workers and MSM in 2017 found that studies measuring stigma used various metrics that were not necessarily developed for the populations of interest and were largely not validated [66]. Additionally, most stigma measures among MSM addressed stigma based on sexual orientation rather than behaviour, limiting the generalisability of the measures to other settings where understandings of sexual orientation and identities may differ. Additional estimates of prevalence that reflect the different exposure histories are needed. These could come from cross-sectional studies and population surveillance exploring exposure over different recall periods. Furthermore, rates of exposure from longitudinal studies would be useful to inform models. In the absence of these, or if estimates from longitudinal studies may be limited (e.g. if there is substantial loss-to-follow-up), rates could be estimated by fitting the model to good quality cross-sectional data measured at different time points.

Despite increasing recognition of the importance of structural determinants for HIV transmission, estimation of the total effect of structural determinants on HIV outcomes has generally been overlooked in epidemiological analyses, except for socioeconomic status (e.g. income, education, employment) [67–69]. Previously, many estimates have been based on cross-sectional studies and ecological analyses, which despite being useful, may have limited value for causal inferences given the risk for reverse causation, confounding, and ecological fallacy [70]. To improve the strength of evidence linking structural determinants, mediators, and HIV outcomes, causal analyses of longitudinal studies are needed (Fig. 3b). A challenge is the potential abundance of confounding factors that may or may not be measured, but which may need to be adjusted for [71]. Ignoring this background heterogeneity could risk biasing the contribution of the structural determinant to HIV outcomes in the model. Empirical evidence (e.g. reviews of quantitative studies) can help identify the confounders to consider and directed acyclic graphs (DAGs) can help choose which to control for [72]. Estimates from path-specific inferences such as causal mediation analyses could be used to parameterise effect sizes [73]. Mediation analyses can be used to estimate causal estimands of exposure to structural determinants, including natural direct and indirect effects, path-specific effects, controlled direct effects, and proportions mediated, using longitudinal data (Additional file 1: Text S3) [74]. To improve the validity of model predictions, effect sizes should ideally be based on the same exposure definitions and settings as the other parameters (e.g. proportions exposed, exposure rates) that inform the model.

Although it may not be possible to randomise (at the individual or cluster-level) some structural determinants (e.g. criminalisation), evidence on the causal effects and impacts of structural interventions should ideally come from randomised controlled trials (RCTs)—often considered the gold standard for causal inference analyses (Fig. 3b). For example, there have been several RCTs of individual and community-level interventions to address inequitable gender norms [75–82]. However, even with RCTs, additional analyses might be needed to identify and quantify specific causal pathways. For example, RCT data has also been used in causal mediation analyses to estimate the effects of exposure to interventions on inequitable gender norms along specific pathways [83, 84].

Given the challenges associated with obtaining causal estimates, evidence on structural determinants and causal pathways should be complemented with information from additional sources, including qualitative evidence, social theory, and inputs and involvement in the research from people with lived experience, ideally from the same or similar settings and populations as the ones modelled [85]. In our review, 11 of the studies that modelled specific settings included co-authors from those settings; however, it was generally unclear if people with lived experienced from those settings were involved in the studies. Finally, modellers should aim for transparency in reporting the strengths of evidence on model assumptions related to structural determinants and attempt to triangulate all relevant data to help identify and quantify sources of uncertainty using distributions of parameter values.

## Discussion

In this paper, we introduce conceptual and methodological frameworks to assist investigations of the population-level impacts of structural determinants on HIV outcomes, underpinned by a scoping review of previous models. Simultaneously, we advocate for strengthening the empirical evidence of the effects of structural determinants and interventions on HIV outcomes—an essential foundation for developing better models and prioritising interventions.

Previous models of structural determinants and interventions include notable efforts to represent structural determinants dynamically, with particularly complex representations of violence and incarceration, which were modelled in several studies with multiple exposures, exposure histories, and additional stratifications. Our recommendations aim to build upon these to help the next generation of models represent structural determinants dynamically and mechanistically and to portray the important causal pathways and mediators to produce useful, evidence-based estimates of the impacts of structural determinants and interventions. These insights could be useful to inform policy decisions for resource allocation [86]. Further, our methodological framework supports transparency in reporting of methods and assumptions to facilitate comparisons in approaches and results across studies, which differed among the studies identified in our review.

Others have considered how to represent social and structural determinants in transmission dynamic models of infectious diseases [71, 87, 88]. Although our framework was principally developed to support the design of HIV models, our recommendations have broad applicability and can be readily extended to models of other infectious diseases that may face similar limitations. Indeed, a previous review of tuberculosis models also found few models that represented structural determinants (e.g. undernutrition, wealth), which were limited by simple exposure representations and causal pathways, an almost exclusive focus on proximate structural determinants, and a lack of evidence on the exposures from the necessary contexts [88]. More generally, we advocate a mechanistic approach with an emphasis on understanding and reproducing the key causal pathways, which is adaptable yet applicable to multiple and diverse structural determinants, mediators, and outcomes, in various contexts.

## Conclusions

Increasingly, transmission dynamic models are being used to explore how exposures to structural determinants influence social and health inequalities, and how structural interventions might mitigate these impacts. Models informed by strong evidence on the causal pathways linking structural determinants and interventions to changes in HIV outcomes—through their direct and indirect effects on downstream mediators—can be used to estimate the contribution of structural determinants to HIV epidemics and to predict the impacts of structural interventions. Our recommendations for the next generation of models can help modellers think about how to model exposure to structural determinants and interventions dynamically and mechanistically to improve estimation of their impacts. Future research should prioritise longitudinal studies designed to estimate the causal effects of structural determinants on mediators and HIV over suitable timeframes. This will not only contribute to a deeper understanding of structural determinants, but also facilitate greater use of models in exploring the impacts and economic feasibility of structural interventions, which will be critical in the next phase of the global HIV response.

## Supplementary Information

Additional file 1: Text S1. Additional scoping review methods. Text S2. Additional results of scoping review. Text S3. Definitions of effects that can be estimated using causal mediation analysis.

Additional file 2: Table S1. Examples of structural determinants, societal enablers, and structural interventions identified in the UNAIDS Global AIDS Strategy 2021-2026 that are important for HIV transmission, and the mechanisms through which they impact HIV. Table S2. Scoping review search terms and hits. Table S3. Preferred Reporting Items for Systematic reviews and Meta-Analyses extension for Scoping Reviews (PRISMA-ScR) Checklist. Table S4. Additional information on the modelling of structural determinants and interventions in the studies identified in the scoping review. Table S5. Empirical evidence used to parameterise models of exposure to structural determinants. Table S6. Data on HIV epidemiology and structural determinants used to calibrate the models.

Additional file 3: Figure S1. PRISMA-ScR checklist for the scoping review.

## Data Availability

All information extracted and analysed during this study is included in this published article (and its supplementary information files).

## Abbreviations

ART: Antiretroviral therapy
AIDS: Acquired immune deficiency syndrome
FSW: Female sex workers
HIV: Human immunodeficiency virus
MSM: Men who have sex with men
PAF: Population attributable fraction
PLHIV: People living with HIV
PrEP: Pre-exposure prophylaxis
PWID: People who inject drugs
R_0_: Basic reproduction number
STI: Sexually transmitted infection
tPAF: Transmission population attributable fraction
UNAIDS: Joint United Nations Programme on HIV/AIDS
