# Supplementary material for: From conceptualising to modelling structural determinants and interventions in HIV transmission dynamics models: a scoping review and methodological framework for evidence-based analyses
Source: BMC Med. 2024 Sep 19;22:404. doi: 10.1186/s12916-024-03580-z (PMC11414142; doi:10.1186/s12916-024-03580-z)
Supplement: Supplementary file 1 — Additional file 1: Text S1. Additional scoping review methods. Text S2. Additional results of scoping review. Text S3. Definitions of effects that can be estimated using causal mediation analysis. [file 12916_2024_3580_MOESM1_ESM.docx]

**Additional file 1: Supplementary text**

**Text S1. Additional scoping review methods**

*Search, screening, and data extraction*

We first screened studies by title and abstract, then screened full texts for eligible studies. We included peer-reviewed studies that used transmission dynamic models (i.e., models in which the force of infection varies as a function of the prevalence of infection and therefore time)[26] to estimate the impacts of structural determinants and/or interventions on HIV transmission, in any population and setting. We excluded studies that did not model structural determinants or HIV, were “static” statistical models, did not estimate the impact of structural factors on HIV transmission (e.g., modelled incarceration but did not estimate its impact), that only modelled scale-up of biomedical interventions (e.g., pre-exposure prophylaxis (PrEP), antiretroviral therapy (ART)) or that were not in English.

From the included studies, we extracted information on key model characteristics including the type of model (i.e., compartmental or agent-based), year of publication, the populations modelled, and information on 1) whether structural factors were represented statically or dynamically, and whether they stratified by exposure history, 2) the mediators linking exposure to the structural determinant to HIV outcomes, 3) data related to the structural factors used to parameterise and calibrate the models, and 4) the main outcomes modelled and how impacts were estimated (i.e., modelling scenario definitions). We used this information to appraise how structural determinants and/or interventions were modelled, and what information and changes could improve future modelling of structural determinants.

Screening and data extraction were conducted by JS independently. Discrepancies were resolved by MM-G and M-CB.

**Text S2: Additional results of scoping review**

*Search results*

We identified 2510 publications, removed 401 duplicates and 2031 titles at the title and abstract screening stage, then assessed the eligibility of 78 full texts (Additional file 3: Figure S1). Of these, we included 17 unique studies that used 13 unique models to assess the impact of structural factors and/or interventions on HIV transmission.

**Text S3: Definitions of effects that can be estimated using causal mediation analysis**[74]

**Controlled direct effect:** How much the HIV acquisition risk would have changed if everyone experienced the same level of a specific mediator, e.g., if everyone used condoms.

**Natural direct effect:** How much of the HIV acquisition risk due to a structural determinant is not mediated by a specific mediator, i.e., the effect of exposure through pathways that do not contain the mediator. As such it may represent both an actual direct effect of exposure on HIV acquisition risk, as well as indirect effects through unobserved or unmeasured mediators.

**Natural indirect effect:** How much of the HIV acquisition risk due to exposure to a structural determinant is mediated by a specific mediator, e.g., condom use.

**Path-specific effect:** How much of the HIV acquisition risk due to a structural determinant is mediated by an additional mediator (e.g., non-viral suppression of the male partner), beyond mediation by the first mediator (condom use).

**Proportion mediated:** The proportion of the total effect that is mediated through a specific mediator (or combination of mediators).
