# Supplementary material for: From conceptualising to modelling structural determinants and interventions in HIV transmission dynamics models: a scoping review and methodological framework for evidence-based analyses
Source: BMC Med. 2024 Sep 19;22:404. doi: 10.1186/s12916-024-03580-z (PMC11414142; doi:10.1186/s12916-024-03580-z)
Supplement: Supplementary file 2 — Additional file 2: Table S1. Examples of structural determinants, societal enablers, and structural interventions identified in the UNAIDS Global AIDS Strategy 2021-2026 that are important for HIV transmission, and the mechanisms through which they impact HIV. Table S2. Scoping review search terms and hits. Table S3. Preferred Reporting Items for Systematic reviews and Meta-Analyses extension for Scoping Reviews (PRISMA-ScR) Checklist. Table S4. Additional information on the modelling of structural determinants and interventions in the studies identified in the scoping review. Table S5. Empirical evidence used to parameterise models of exposure to structural determinants. Table S6. Data on HIV epidemiology and structural determinants used to calibrate the models. [file 12916_2024_3580_MOESM2_ESM.docx]

**Additional file 2: Supplementary tables**

**Table S1. Examples of structural determinants, societal enablers, and structural interventions identified in the UNAIDS Global AIDS Strategy 2021-2026 that are important for HIV transmission, and the mechanisms through which they impact HIV.** Note that this is not a definitive list, and that further research is needed to determine the importance of these factors, and to determine and confirm the mechanisms, mediators, and pathways by which they influence HIV outcomes.[10]

| Structural determinant | Populations potentially impacted | Example societal enablers or structural interventions | Example mechanisms of impacting HIV outcomes |
| --- | --- | --- | --- |
| Stigma and discrimination | MSM, transgender and non-binary people, FSW, PWID, PLHIV, women & girls | Anti-discrimination laws. Public awareness campaigns. Sensitivity training for health care workers. Access to HIV self-testing. Integrating other social and health services (e.g., gender affirming care) into HIV services. Community mobilisation and peer support programmes. | Reduced access to HIV testing, treatment, and prevention. Mental health outcomes (depression, anxiety, low self-esteem). Substance use as a coping mechanism. Sexual behaviours. |
| Gender inequalities and gender-based violence | Women & girls, FSW, transgender and non-binary people | Investments in social protection and education for women & girls | Reduced access to education, economic opportunities, and health care. Inability to negotiate safe sex practices. Intimate partner violence, including sexual violence. |
| Punitive laws and policies | MSM, transgender and non-binary people, PWID, FSW, PLHIV, women & girls | Decriminalisation of same-sex behaviours, drug use, and sex work. Gender self-identification laws. | Reduced access to HIV testing, treatment, and prevention due to fear of legal repercussions. Lack of availability of harm reduction services, such as needle and syringe exchange programmes and opioid agonist therapy. |
| Poverty & inadequate living conditions | All | Universal health coverage. Integrated food and nutrition programmes and social protection interventions. Microfinancing interventions. | Reduced access to HIV testing, treatment, and prevention due to prioritising basic needs (e.g., food) over health care, which may be expensive. Late diagnosis and treatment. Survival sex work. |
| FSW=female sex workers, MSM=men who have sex with men, PLHIV=people living with HIV, PWID=people who inject drugs | | | |

**Table S2a. Medline scoping review search terms and hits.** The search was conducted on Monday August 28^th^, 2023.

|  | **Hits** | **Search term** |
| --- | --- | --- |
| 1 | 107772 | exp HIV/ |
| 2 | 316769 | exp HIV Infections/ |
| 3 | 78683 | exp Acquired Immunodeficiency Syndrome/ |
| 4 | 534644 | (HIV OR HIV1* OR HIV2* OR HIV-1* OR HIV-2*).af |
| 5 | 109478 | (human immun#deficiency virus OR human immun# deficiency virus).af |
| 6 | 99687 | (acquired immun#deficiency syndrome OR acquired immun# deficiency syndrome).af |
| 7 | 470051 | OR/ 1-6 |
| 8 | 357009 | Models, Biological/ |
| 9 | 157616 | Models, Theoretical/ |
| 10 | 211229 | Computer Simulation/ |
| 11 | 1392 | Patient-Specific Modeling/ |
| 12 | 32326 | Monte Carlo Method/ |
| 13 | 31138 | exp Stochastic Processes/ |
| 14 | 261917 | ((math* OR transmission OR dynamic* OR epidemi* OR compartmental OR deterministic OR individual OR individual#based OR agent OR agent#based OR network OR simulat*) ADJ3 model*).af |
| 15 | 856907 | OR/ 8-14 |
| 16 | 512494 | exp Socioeconomic Factors/ |
| 17 | 100 | Socioeconomic disparities in health/ |
| 18 | 19784 | exp health status disparities/ |
| 19 | 9845 | Ill-Housed Persons/ |
| 20 | 112973 | exp Violence/ |
| 21 | 18365 | Prisoners/ |
| 22 | 43876 | Poverty/ |
| 23 | 12872 | exp Social Discrimination/ |
| 24 | 12899 | Social Stigma/ |
| 25 | 107730 | ((structural OR social) ADJ3 (determinant* OR factor* OR condition* OR cause* OR enabler* OR driver* OR exposure* OR risk*)).af |
| 26 | 412263 | (criminali#ation OR homeless* OR unstable housing OR housing instability OR incarceration OR prison* OR stigma OR discrimination OR violence OR poverty).af |
| 27 | 988692 | OR/ 16-26 |
| **28** | **619** | **AND/ 7 & 15 & 27** |

**Table S2b. Embase scoping review search terms and hits.** The search was conducted on Monday August 28^th^, 2023.

|  | **Hits** | **Search term** |
| --- | --- | --- |
| 1 | 220799 | exp Human immunodeficiency virus/ |
| 2 | 426120 | exp Human immunodeficiency virus infection/ |
| 3 | 154802 | exp acquired immune deficiency syndrome/ |
| 4 | 491559 | (HIV OR HIV1* OR HIV2* OR HIV-1* OR HIV-2*).af |
| 5 | 512168 | (human immun#deficiency virus OR human immun# deficiency virus).af |
| 6 | 152857 | (acquired immun#deficiency syndrome OR acquired immun# deficiency syndrome).af |
| 7 | 655123 | OR/ 1-6 |
| 8 | 140638 | Mathematical model/ |
| 9 | 208325 | Biological model/ |
| 10 | 96769 | Theoretical model/ |
| 11 | 140067 | Computer simulation/ |
| 12 | 7316 | Population model/ |
| 13 | 208325 | Biological model/ |
| 14 | 22033 | Stochastic model/ |
| 15 | 387279 | ((math* OR transmission OR dynamic* OR epidemi* OR compartmental OR deterministic OR individual OR individual#based OR agent OR agent#based OR network OR simulat*) ADJ3 model*).af |
| 16 | 800717 | OR/ 8-15 |
| 17 | 1360451 | exp socioeconomics/ |
| 18 | 86398 | exp social aspect/ |
| 19 | 34630 | exp health disparity/ |
| 20 | 13508 | homelessness/ |
| 21 | 180604 | exp Violence/ |
| 22 | 2725 | Correctional facility/ |
| 23 | 57020 | Poverty/ |
| 24 | 34558 | exp Social Discrimination/ |
| 25 | 14790 | Social Stigma/ |
| 26 | 127139 | ((structural OR social) ADJ3 (determinant* OR factor* OR condition* OR cause* OR enabler* OR driver* OR exposure* OR risk*)).af |
| 27 | 525790 | (criminali#ation OR homeless* OR unstable housing OR housing instability OR incarceration OR prison* OR stigma OR discrimination OR violence OR poverty).af |
| 28 | 2041494 | OR/ 16-27 |
| **29** | **1891** | **AND/ 7 & 16 & 28** |

**Total from both databases = 2510**

**Total after removing duplicates = 2109**

**Duplicates = 401**

**Table S3. Preferred Reporting Items for Systematic reviews and Meta-Analyses extension for Scoping Reviews (PRISMA-ScR) Checklist**

| **SECTION** | **ITEM** | **PRISMA-ScR CHECKLIST ITEM** | **REPORTED IN** |
| --- | --- | --- | --- |
| **TITLE** | | | |
| Title | 1 | Identify the report as a scoping review. | Title |
| **ABSTRACT** | | | |
| Structured summary | 2 | Provide a structured summary that includes (as applicable): background, objectives, eligibility criteria, sources of evidence, charting methods, results, and conclusions that relate to the review questions and objectives. | Abstract |
| **INTRODUCTION** | | | |
| Rationale | 3 | Describe the rationale for the review in the context of what is already known. Explain why the review questions/objectives lend themselves to a scoping review approach. | Introduction (background) |
| Objectives | 4 | Provide an explicit statement of the questions and objectives being addressed with reference to their key elements (e.g., population or participants, concepts, and context) or other relevant key elements used to conceptualize the review questions and/or objectives. | Introduction (objectives) |
| **METHODS** | | | |
| Protocol and registration | 5 | Indicate whether a review protocol exists; state if and where it can be accessed (e.g., a Web address); and if available, provide registration information, including the registration number. | Not applicable |
| Eligibility criteria | 6 | Specify characteristics of the sources of evidence used as eligibility criteria (e.g., years considered, language, and publication status), and provide a rationale. | Scoping review |
| Information sources* | 7 | Describe all information sources in the search (e.g., databases with dates of coverage and contact with authors to identify additional sources), as well as the date the most recent search was executed. | Scoping review, Additional file 2: Table S2) |
| Search | 8 | Present the full electronic search strategy for at least 1 database, including any limits used, such that it could be repeated. | Additional file 2: Table S2 |
| Selection of sources of evidence† | 9 | State the process for selecting sources of evidence (i.e., screening and eligibility) included in the scoping review. | Additional file 1: Text S1 |
| Data charting process‡ | 10 | Describe the methods of charting data from the included sources of evidence (e.g., calibrated forms or forms that have been tested by the team before their use, and whether data charting was done independently or in duplicate) and any processes for obtaining and confirming data from investigators. | Additional file 1: Text S1 |
| Data items | 11 | List and define all variables for which data were sought and any assumptions and simplifications made. | Additional file 1: Text S1, Additional file 2: Table S3 |
| Critical appraisal of individual sources of evidence§ | 12 | If done, provide a rationale for conducting a critical appraisal of included sources of evidence; describe the methods used and how this information was used in any data synthesis (if appropriate). | Scoping review, Additional file 1: Text S1 |
| Synthesis of results | 13 | Describe the methods of handling and summarizing the data that were charted. | Scoping review, Additional file 1: Text S1 |
| **RESULTS** | | | |
| Selection of sources of evidence | 14 | Give numbers of sources of evidence screened, assessed for eligibility, and included in the review, with reasons for exclusions at each stage, ideally using a flow diagram. | Additional file 3: Figure S1 |
| Characteristics of sources of evidence | 15 | For each source of evidence, present characteristics for which data were charted and provide the citations. | Table 1, Additional file 2: Table S3 |
| Critical appraisal within sources of evidence | 16 | If done, present data on critical appraisal of included sources of evidence (see item 12). | Scoping review (structural determinants and interventions examined, representations, use of empirical data) |
| Results of individual sources of evidence | 17 | For each included source of evidence, present the relevant data that were charted that relate to the review questions and objectives. | Table 1, Additional file 2: Tables S3-5 |
| Synthesis of results | 18 | Summarize and/or present the charting results as they relate to the review questions and objectives. | Scoping review (structural determinants and interventions examined, representations, use of empirical data), Table 1, Additional file 2: Tables S3-5 |
| **DISCUSSION** | | | |
| Summary of evidence | 19 | Summarize the main results (including an overview of concepts, themes, and types of evidence available), link to the review questions and objectives, and consider the relevance to key groups. | Discussion |
| Limitations | 20 | Discuss the limitations of the scoping review process. | Discussion |
| Conclusions | 21 | Provide a general interpretation of the results with respect to the review questions and objectives, as well as potential implications and/or next steps. | Discussion |
| **FUNDING** | | | |
| Funding | 22 | Describe sources of funding for the included sources of evidence, as well as sources of funding for the scoping review. Describe the role of the funders of the scoping review. | Acknowledgements and declarations |

JBI = Joanna Briggs Institute; PRISMA-ScR = Preferred Reporting Items for Systematic reviews and Meta-Analyses extension for Scoping Reviews.

* Where *sources of evidence* (see second footnote) are compiled from, such as bibliographic databases, social media platforms, and Web sites.

† A more inclusive/heterogeneous term used to account for the different types of evidence or data sources (e.g., quantitative and/or qualitative research, expert opinion, and policy documents) that may be eligible in a scoping review as opposed to only studies. This is not to be confused with *information sources* (see first footnote).

‡ The frameworks by Arksey and O’Malley (6) and Levac and colleagues (7) and the JBI guidance (4, 5) refer to the process of data extraction in a scoping review as data charting*.*

§ The process of systematically examining research evidence to assess its validity, results, and relevance before using it to inform a decision. This term is used for items 12 and 19 instead of "risk of bias" (which is more applicable to systematic reviews of interventions) to include and acknowledge the various sources of evidence that may be used in a scoping review (e.g., quantitative and/or qualitative research, expert opinion, and policy document).

*From:* Tricco AC, Lillie E, Zarin W, O'Brien KK, Colquhoun H, Levac D, et al. PRISMA Extension for Scoping Reviews (PRISMAScR): Checklist and Explanation. Ann Intern Med. 2018;169:467–473. [doi: 10.7326/M18-0850](http://annals.org/aim/fullarticle/2700389/prisma-extension-scoping-reviews-prisma-scr-checklist-explanation).

**Table S4. Additional information on the modelling of structural determinants and interventions in the studies identified in the scoping review.**

| **Reference** | **Type of model** | **Population** | **Structural determinants and/or intervention** | **Objectives** | **Details of exposure to structural determinants and interventions** | **Key causal pathways, mediators, and assumptions** |
| --- | --- | --- | --- | --- | --- | --- |
| **a) Static approaches to representing exposure to structural determinants** | | | | | | |
| Stover et al., 2021[55] | Compartmental (Goals) | Heterosexual men and women, FSW, MSM, and PWID | UNAIDS 10-10-10 (Decriminalisation of sex work and drug use, removing internalised HIV stigma, eliminating gender-based violence against women) | Estimate the impact of achieving the UNAIDS 2025 targets | Internalised stigma modelled by estimating maximum treatment cascade targets achievable without addressing stigma and applying these lower cascade targets to all countries.  Access to justice modelled by applying set reduction in new infections among FSW over 10 years and attributing the reduction to access to justice.  Violence modelled as reduction in linkage to HIV care and ART adherence.  No movement between exposed and unexposed states but coverage (% exposed) was varied in modelling scenarios. | Societal enablers assumed to impact HIV indirectly (stigma and violence reduction) and directly (decriminalisation).  Internalised stigma assumed to ↓ HIV testing, ART initiation, and adherence. Access to justice assumed to ↓ HIV transmission. Violence assumed to ↓ linkage to care and ART adherence. |
| Levy et al., 2021[51] | Compartmental | Heterosexual men and women | Internalised, enacted, and perceived HIV stigma and stigma reduction | Predict reductions in HIV infection through potential interventions that alter stigma over time. | Stigma modelled using parameter that changes over time, representing the proportion of the population with stigmatising views. | Stigma assumed to impact HIV indirectly through ↓ rates of ART use (uptake and discontinuation) among PLHIV. |
| Ronoh et al.,2020[58] | Compartmental | Heterosexual men and women aged 15-24 | Positive and negative attitudes^a^ | Estimate the effects of varying HIV testing, condom use, and ART adherence on HIV among youth in Kenya exposed to attitudes influencing disease control. | Positive and negative attitudes modelled as proportions that influence model parameters. | Positive and negative attitudes assumed to impact HIV indirectly: ↑ positive attitudes ↑ rates of condom use, HIV testing, and ART use. ↑ negative attitudes ↓ these rates. |
| Vassall et al., 2014[56] | Compartmental | FSW | Community mobilisation and empowerment for FSW | Estimate the cost-effectiveness of community mobilisation and empowerment interventions in the Avahan programme in India | Community mobilisation and empowerment modelled by assuming that a fraction of the total increase in condom use due to Avahan was due to community mobilisation and empowerment, based on empirical analyses, removing this fraction in simulations, and attributing the reduction to community mobilisation and empowerment. | Community mobilisation and empowerment assumed to ↑ FSW condom use with clients. |
| Wirtz et al., 2014[57] | Compartmental (Goals) | Heterosexual men and women, including FSW) | Community empowerment for FSW | Estimate the impact of scale-up of a community empowerment intervention among FSW | Modelled as the proportion of the population exposed. No movement between states, but coverage (% exposed) was varied in modelling scenarios. | Empowerment assumed to impact HIV indirectly through  ↑ condom use and ↑ effectiveness of ART among FSW exposed to empowerment intervention. |
| Decker et al., 2013[49] | Compartmental (Goals) | FSW and non-FSW (gender-stratified)) | Violence against FSW and reducing violence | Estimate the impact of reducing violence against FSWs on HIV epidemics in Ukraine and Kenya | Modelled as the proportion of FSW exposed to violence. | Violence among FSW assumed to impact HIV indirectly through ↓ condom use during vaginal sex and ↓↓ condom use during anal sex among FSW exposed to violence and ↑ HIV transmission probability through condomless anal than vaginal sex. |
| Strathdee et al., 2010[5] | Compartmental | PWID, including heterosexual men and women, bisexual MSM, and exclusive MSM ^b^ | Elimination of police beatings in Ukraine and scale-up of OAT, NSPs, and ART | Characterise how certain structural changes could potentially affect proximate risk determinants and influence HIV epidemics among PWID | Beatings not explicitly modelled, but a scenario with reduced sharing of injection equipment based on empirical analyses of reduced sharing if ever exposed to violence was modelled, and the reduction was assumed to be attributed to eliminating violence.  **Additional stratifications:**  OAT and NSP status (currently, not on OAT/NSPs) | Beatings assumed to impact HIV indirectly through ↑ sharing of non-sterile injection equipment. |
| **b) Stratification-based approaches to representing structural determinants, where the modelled population could experience one level of exposure, with some movement between exposed and non-exposed states** | | | | | | |
| Rigby and Johnson 2017[52] | Individual-based | Heterosexual men and women | Intimate partner violence against women and violence reduction based on two interventions (IMAGE and SASA!) | Identify causal pathways and confounders that play an important role in the IPV-HIV relationship and estimate which interventions can reduce HIV incidence by reducing IPV. | IPV = sexual or physical violence, at partnership level.  **No. IPV states:** 2 (no IPV in partnership, IPV in partnership). Once there is IPV, partnerships remain violent for their duration.  **Movement between states:** yes, one-way (no IPV 🡪 IPV).  **Additional stratifications considered:**  **Partnership level:** partnership type (married <2 years, married >2 years, short-term, sex worker-client. IPV can only occur only in married and short-term partnerships).  **Individual level:**  **Men and women:** sexual behaviour group (high risk [concurrent and sex-worker client partnerships possible] vs low risk [monogamous only]).  **Men:** predisposition for violence (violent vs not; IPV can only occur if man predisposed to violence. ↑probability of being violent if high risk).  **Women:** “susceptibility factor” (number 0-1 randomly assigned to women to account for extra heterogeneity e.g., due to self-esteem, mental health. ↑susceptibility = ↑IPV rate). | IPV affects HIV indirectly.  Mediators were varied in different model scenarios that explored different causal pathways.  **Mediators^c^:**  ↓condom use, ↑relationship dissolution, ↓marriage rate (short-term relationships), ↑secondary partners (women only), and ↓viral suppression (women only), in violent partnerships. Men and women in violent partnerships therefore both have ↑HIV acquisition risk. |
| Stone et al., 2022[53] | Compartmental | PWID (not gender-stratified) | Housing instability among PWID | Estimate global and national % of incident HIV among PWID due to housing instability | **No. unstable housing states:** 2 (stably, unstably housed in the past year).  **Movement between states:** yes. Fixed rate determined by average duration PWID are unstably housed. | Unstable housing impacts HIV directly.  ↑ HIV transmission risk if unstably housed. |
| **c) Stratification-based approaches to representing structural determinants with multiple exposure histories** | | | | | | |
| Shannon et al., 2015[4] | Compartmental | FSW and clients | Violence against FSW and various hypothetical interventions including elimination of sexual violence, decriminalisation of sex work, increasing safer sex work environments, community empowerment and outreach | Estimate infections averted through structural changes in regions with concentrated and generalised epidemics, and high HIV prevalence among FSW | **No. violence states:**  **Canada:** 6 (never, recent (<6 months) and non-recent (>6 months) police harassment, recent (<6 months) and non-recent (>6 months) physical violence, ever client sexual violence).  **India:** 5 (never, recent <12 months) and non-recent (>12 months) client violence, recent (<6 months) and non-recent (>6 months) fear of condom confiscation).  **Kenya:** 3 (never, recent (<12 months) client sexual violence, non-recent (>12 months) client sexual violence).  **Movement between violence states:** yes. In Canada, recent client physical violence must occur before client sexual violence. Once in ever client sexual violence compartment, FSW remain there.  **No. work environment states:**  3. FSW assigned to 1 of 3 fixed work environments, from least to most safe. Options differ by setting.  **Canada:** street, informal indoor venues, formal sex work establishments.  **India:** home, street, brothel.  **Kenya:** bar, street, home.  **Movement between work environment states:** no. Work environments were fixed in the baseline scenario (although transitions were possible in other modelling scenarios).  **Additional stratifications considered:**  **Canada only:** PWID status (FSW who ever injected drugs have ↓ condom use).  **India only:** sex worker collectivisation (members of sex work collectives have ↑ condom use).  **Kenya only:** binge drinking (FSW who binge drink have ↑ rate of sexual violence, ↑ number of clients, and ↓ condom use) | Violence affects HIV indirectly.  **Mediators:**  **All settings:** ↑ condom use and ↓ risk of violence in safer worker environments.  **Canada:** recent police harassment, recent and non-recent client physical violence, and ever client sexual violence ↓ condom use. ↑risk of recent police harassment if exposed to recent client physical violence, and vice versa. No effect of non-recent police harassment on condom use.  **India:** recent client violence and recent fear of condom confiscation ↓ condom use. No effect of non-recent client violence or non-recent fear of condom confiscation on condom use.  **Kenya:** recent client sexual violence ↓condom use. No effect of non-recent client sexual violence on condom use. A fraction of all FSW is exposed to FSW outreach, which ↑condom use. |
| Ward et al., 2022[54] | Compartmental | PWID (not gender-stratified) | Incarceration of PWID and drug law reform | Estimate the cost-effectiveness of shift from criminalising drug users to a public health approach with scale up of OAT and ART | **No. incarceration states:** 4 (never, current, recent (<6 months), non-recent (>6 months).  **Movement between states:** yes. ↑ incarceration rate if previously incarcerated.  **Additional stratifications:**  OAT status (↓ reincarceration rate if on OAT), current and ex-injectors (only current PWID experience incarceration). | Incarceration affects HIV directly.  ↑transmission risk among PWID recently released than never or non-recently incarcerated. Transmission risk while currently incarcerated can be ↑ or ↓ depending on setting. |
| Adams et al., 2021[44] | Individual-based (TITAN model) | African American men and women. Only men can be incarcerated. | Incarceration of African American men and different PrEP prescription strategies for women with incarcerated male partners | Estimate the potential reduction in HIV transmission among women attributable to making PrEP accessible to women affected by partner incarceration. | Same as above. Incarceration rates are also higher for men living with HIV and current PWID. | Same as above. Incidence and prevalence are also higher among MSMW. |
| Bernard et al., 2020[46] | Individual-based (network model) | PWID, people who use drugs, MSM, and lower-risk heterosexuals (gender-stratified) | Incarceration of PWID and jail diversion program for low-level drug offenders | To assess the health benefits and cost-effectiveness of a jail diversion program for low-level drug offenders | **No. incarceration states:** 5 (currently in drug court, currently incarcerated in jail or prison, currently in diversion program, or not incarcerated). Men and women.  **Movement between states:** yes.  **Additional stratifications:** type of crime (misdemeanor vs felony; misdemeanor = variable length jail stay, felony = jail prior to trial followed by release, jail, or prison. If misdemeanor PWID can enroll in diversion program, otherwise enter jail), jail further stratified by whether awaiting court proceeding or serving sentence. | Incarceration impacts HIV indirectly.  No HIV transmission in jail. Post-release, PWID less likely to be in NSPs, SUDT, and ART. Jail diversion program and drug court ↓ % of PWID, which ↓ HIV transmissions. |
| Adams et al., 2018[45] | Individual-based (TITAN model) | African American men and women. Only men can be incarcerated. | Incarceration of African American men | Determine which mediators of male incarceration are most important for HIV acquisition among women, which could be targets for intervention. | **No. of incarceration states:** 4 (never, currently incarcerated, recently released (*<6 months*) and non-recently released (*>6 months*)). Men only.  **Movement between states:** yes. ↑ incarceration rates if previously incarcerated  **Additional stratifications considered:** type of facility (↓ incarceration rates and ↑ duration of current incarceration in prisons vs jails). Incarceration rates and durations were fixed over time. | Incarceration affects HIV indirectly.  **Mediators:**  **Men**: ↑ probability of relationship dissolution while incarcerated. ↑ number of sexual partners, ↑ probability of current STI, and ↑ probability of ART dropout for all men recently released.  **Women** (*only applies if main partner incarcerated*): a fraction have ↑ number of sexual contacts throughout partner’s incarceration or for 6 months after the relationship ends, if it ends whilst he is incarcerated. |
| Borquez et al., 2018[48] | Compartmental | PWID (gender-stratified) | Incarceration of PWID and syringe confiscation by police and drug law reform that institutes drug treatment instead of incarceration, compulsory abstinence programme | To investigate the past and future effect of drug law reform in 2012 that instituted drug treatment instead of incarceration on HIV incidence | **No. incarceration states:** 4 (never, current, recent (<6 months), non-recent (>6 months). Men and women.  **Movement between states**: yes. ↑ incarceration rates if previously incarcerated  **No. syringe confiscation states:** 2 (syringe confiscation in the past 6 months, no syringe confiscation in the past 6 months)  **Additional stratifications:** exposure to drug treatment (OAT) or rehabilitation (compulsory abstinence programs, CAP). | Incarceration and syringe confiscation affect HIV directly.  ↑ HIV transmission risk among PWID recently incarcerated and with recent syringe confiscation. No interaction between structural determinants. |
| Altice et al., 2016[47] | Compartmental | PWID (not gender-stratified) | Incarceration of PWID and stopping incarceration of PWID and scale-up of prison-based opioid agonist therapies | Assess the long-term contribution of incarceration to HIV transmission among PWID and the impact of eliminating incarceration and scaling up prison-based OAT | **No. incarceration states:** 4 (never, current, recent (<12 months), and non-recent (>12 months)).  **Movement between states:** yes. ↑ incarceration rate if previously incarcerated.  **Additional stratifications:** OAT status (on OAT vs off OAT) | Incarceration affects HIV directly.  ↑ HIV acquisition rate among PWID previously (recently and non-recently) than never or currently incarcerated; ↑↑ acquisition rate among PWID recently than non-recently incarcerated. (implicitly assumed ↑ is due to ↑ frequency of sharing injection equipment). OAT assumed to ↓HIV infectivity and susceptibility by 50% (mechanism unspecified). |
| Dolan et al., 2016[50] | Compartmental | PWID and non-PWID (not gender-stratified) | Incarceration of PWID and reduced incarceration, scale-up of prison-based and post-release OAT, retention on ART post-release | Model the contribution of incarceration to HIV incidence in PWID and examine the effects of reduced incarceration, prison-based OAT, and post-release ART retention | **No. incarceration states:** 4 (never, current, recent (<6 months), and non-recent (>6 months))  **Movement between states:** yes.  **Additional stratifications:** Sharing status (non-PWID, PWID who do not share syringes [never sharers & temporary sharers while incarcerated], PWID who share syringes), | Incarceration affects HIV indirectly.  **Mediators:**  Syringe sharing only occurs between PWID who share syringes. ↑↑ % of PWID share syringes whilst currently than recently incarcerated, equal and ↓↓ % among those never & non-recently incarcerated. ↓ART if recently than currently incarcerated.  **Also:** ↓risk of reincarceration and ↓HIV acquisition rate on OAT. |
| ART=antiretroviral therapy, CAP=compulsory abstinence programme, FSW=female sex workers, IPV=intimate partner violence, MSM=men who have sex with men, MSMW=men who have sex with men and women, NSP=needle and syringe programme, PLHIV=people living with HIV, OAT=opioid agonist therapy, PrEP=pre-exposure prophylaxis, PWID=people who inject drugs, STI=sexually transmitted infection, SUDT=substance use disorder treatment, ↑=increases, ↓=decreases. | | | | | | |

**Table S5. Empirical evidence used to parameterise models of exposure to structural determinants.**

| Reference | Parameter | Fixed or calibration-based | Type of empirical evidence used |
| --- | --- | --- | --- |
| 1. Static approaches to representing exposure to structural determinants | | | |
| Stover et al., 2021[55] | Treatment cascade targets in absence of progress on stigma | Fixed | Estimated using evidence from cross-sectional study, nested case-control study, systematic review and meta-analysis |
|  | Reductions in new infections in countries that criminalise sex work and drug injection | Fixed | Estimated from modelling studies |
|  | Reductions in new infections due to a global programme to prevent IPV | Fixed | Estimated from cohort studies, modelling, WHO data |
| Levy et al., 2021[51] | Proportion of the population with stigmatising views of HIV/AIDS in 2003, 2008, and 2014 (based on data on the proportion of women who answered at least two of three questions in a stigmatising manner) | Fixed | Estimated from surveillance data |
| Ronoh et al., 2020[58] | Negative and positive attitude rates influence HIV testing, condom use, and ART | Fixed | Assumed or estimated, source unclear |
| Vassall et al., 2014[56] | Percentage change in condom use due to community mobilisation and empowerment | Fixed | Estimated from Avahan large-scale targeted HIV prevention intervention |
| Wirtz et al., 2014[57] | Impact of empowerment intervention on condom non-use | Fixed | Estimated from WHO reports |
| Decker et al., 2013[49] | Prevalence of violence against FSWs | Fixed | Cross-sectional studies, surveillance data, Sex Worker Advocacy Network (SWAN) report, |
| Strathdee et al., 2010[5] | Reduction in use of non-sterile equipment without police beatings (Ukraine model) | Fixed | Estimated from a cohort study in 3 cities in Ukraine |
| 1. Stratification-based approaches to representing structural determinants, where the modelled population could experience one level of exposure, with some movement between exposed and non-exposed states | | | |
| Rigby and Johnson, 2017[52] | Ratio of probability of violent predispositions in high-risk men vs low-risk men | Fixed | Cross-sectional study |
|  | Probability of violent disposition, high-risk men | Calibration-based | Fitted |
|  | Annual rate of IPV, by relationship duration | Fixed | Fitted |
|  | OR for not using a condom in violent vs non-violent relationships | Fixed | Estimated from cross-sectional study, retrospective cohort study, RCT |
|  | Probability of forced female sexual debut | Fixed | Estimated from surveillance data, cross-sectional studies, and review |
|  | Reduction in marriage rate in violent short-term relationships | Fixed | Assumed |
|  | Increase in rate of relationship dissolution in violent relationships | Fixed | Cohort study |
|  | Increase in rate of acquiring secondary partners among women experiencing IPV | Fixed | Assumed |
|  | Reduction in viral suppression among women on ART experiencing IPV | Fixed | Systematic review and meta-analysis |
| Stone et al., 2022[53] | Relative increase in HIV transmission risk if unstably housed | Calibration-based | Systematic review and meta-analysis of studies globally |
|  | Average duration of unstable housing | Calibration-based | Cohort studies in the US, UK, Canada, Australia |
| 1. Stratification-based approaches to representing structural determinants with multiple exposure histories | | | |
| Shannon et al., 2015[4] | RR of inconsistent condom use due to violence, by violence type and exposure history (Canada model) | Calibration-based | Cohort study |
|  | Time to violence in years by setting, work environment, PWID status, type of violence and exposure history (Canada model) | Calibration-based | Cohort study (Canada) |
|  | Proportion of FSW in different work environments in each setting | Calibration-based | Analysis of IBBA data, cross-sectional studies, systematic review and meta-analysis |
|  | IRR of experiencing recent police harassment if experienced recent client physical violence, vs no police harassment (Canada model) | Calibration-based | Cohort study |
|  | IRR of experiencing recent client physical violence if experienced recent police harassment, vs no client physical violence (Canada model) | Calibration-based | Cohort study |
|  | Time to recent physical and sexual violence (India model) | Calibration-based | Cross-sectional studies |
|  | Time to recent police confiscation, by work environment and sex worker collective status (India model) | Calibration-based | Analysis of IBBA data |
|  | IRR of violence if in sex work collective vs not in collective, by violence type (India model) | Calibration-based | Assumed |
|  | RR of inconsistent condom use due to recent condom confiscation (6 months) and last year client violence (India model) | Calibration-based | IBBA analysis |
|  | IRR of sexual violence if binge drinker (Kenya model) | Calibration-based | Cross-sectional study |
|  | Time to violence if non-binge drinking FSW (Kenya model) | Calibration-based | Cross-sectional study |
|  | RR of inconsistent condom use from binge drinking (Kenya model) | Calibration-based | Cross-sectional study |
|  | RR of inconsistent condom use from recent client sexual violence (Kenya model) | Calibration-based | Cross-sectional studies |
| Ward et al., 2022[54] | Proportion ever incarcerated | Fixed | Cross-sectional studies |
|  | Proportion currently incarcerated | Calibration-based | Assumed |
|  | Average number of times incarcerated if ever incarcerated | Fixed | Cross-sectional surveys |
|  | Average duration of incarceration | Calibration-based | Cross-sectional surveys |
|  | Incarceration and re-incarceration rates | Calibration-based | Fitted |
|  | RR for HIV transmission risk if recently incarcerated compared to not in prison | Calibration-based | Systematic review and meta-analysis |
| Adams et al., 2021[44] | Proportion incarcerated in 2005 | Fixed | Estimated from surveillance data |
|  | Annual probability of incarceration for PWID | Fixed | Surveillance data |
|  | HIV prevalence ratio for incarcerated vs non-incarcerated men | Fixed | Surveillance data |
|  | Rates of incarceration, by type of facility and prior offense | Fixed | Surveillance data |
|  | Sentence lengths, by type of facility and prior offense | Fixed | Surveillance data |
|  | Proportion tested for HIV upon incarceration | Fixed | Surveillance data |
|  | Proportion of PLHIV inmates on ART while incarcerated | Fixed | Systematic review of mostly surveillance data |
|  | Proportion of main relationships that dissolve during incarceration | Fixed | Cross-sectional studies |
|  | Number of partners at start of high-risk period | Calibration-based | Fitted |
|  | Cumulative number of new partners over 6 months | Calibration-based | Fitted |
|  | Increase in HIV acquisition risk due to current STI | Fixed | Cross-sectional study and prospective cohort study |
|  | Proportion of PLHIV inmates retained on ART 6 months post-release | Fixed | Systematic review of mostly surveillance data |
| Bernard et al., 2020[46] | Proportion incarcerated in prison (rather than jail) | Fixed | Surveillance data |
|  | Proportion currently incarcerated, by PWID status, age, sex, ethnicity | Fixed | Surveillance data |
|  | Sentence lengths, by type of facility | Fixed | Surveillance data |
|  | Weekly probability of crime, by age, PWID status, sex, ethnicity | Fixed | Surveillance data |
|  | Fraction of crimes that are felonies, and that result in incarceration or release after trial | Fixed | Surveillance data |
|  | Fraction of misdemeanors that result in transitions to non-drug using population through drug court | Fixed | Surveillance data |
|  | Multiplier for criminal activity if in diversion programme | Fixed | Estimated from non-randomised controlled evaluation |
|  | Multiplier for joining and leaving community programmes if in diversion programme | Calibration-based | Control variable |
|  | Fraction of misdemeanours that result in entry to the diversion programme | Calibration-based | Control variable |
| Adams et al., 2018[45] | Proportion incarcerated in 2005 | Fixed | Estimated from surveillance data |
|  | Rates of incarceration, by type of facility and prior offense | Fixed | Surveillance data |
|  | Sentence lengths, by type of facility and prior offense | Fixed | Surveillance data |
|  | Proportion tested for HIV upon incarceration | Fixed | Surveillance data |
|  | Proportion of PLHIV inmates on ART while incarcerated | Fixed | Systematic review of mostly surveillance data |
|  | Mean number of sex partners for men during 6-months post-release or women with incarcerated partners | Fixed | Longitudinal qualitative study |
|  | Proportion of PLHIV inmates retained on ART 6 months post-release | Fixed | Systematic review of mostly surveillance data |
|  | Probability of ART initiation for those who discontinued post-release | Fixed | Estimated from systematic review of mostly surveillance data |
| Borquez et al., 2018[48] | Proportion exposed to syringe confiscation in the past 6 months at baseline | Calibration-based | Cohort study |
|  | Proportion exited prison in the past 6 months among PWID at baseline | Calibration-based | Cohort study |
|  | Proportion of PWID incarcerated prior to starting injecting | Calibration-based | Fitted |
|  | Primary incarceration rate | Calibration-based | Fitted |
|  | Reincarceration rate | Calibration-based | Cohort study |
|  | Relative change in the proportion of recent receptive sharing among recently released from prison vs never or not recently incarcerated | Calibration-based | Cohort study |
|  | Relative change in the proportion of recent receptive sharing among recently exposed vs unexposed to police syringe confiscation | Calibration-based | Cohort study |
|  | Relative change in the proportion of recent receptive sharing among PWID ever vs never exposed to compulsory abstinence program | Calibration-based | Cohort study |
|  | RR of injecting HIV acquisition among PWID on OAT vs no OAT | Calibration-based | Systematic review and meta-analysis |
|  | Rate of OAT cessation | Fixed | Cohort study, modelling |
| Altice et al., 2016[47] | Duration of incarceration | Calibration-based | Cross-sectional study |
|  | Proportion initiating injecting, by incarceration exposure history (informed by single estimate of proportion of people never incarcerated prior to injecting) | Calibration-based | Cross-sectional study |
|  | Incarceration and re-incarceration rates | Calibration-based | Fitted |
| Dolan et al., 2016[50] | Proportion incarcerated, by PWID status | Calibration-based | Surveillance data |
|  | Proportion of PWID who share syringes in and out of prison | Calibration-based | Reviews, modelling |
|  | Annual number of injections among incarcerated and non-incarcerated PWID | Calibration-based | Modelling, cross-sectional studies, cohort study, cross-over experimental study, |
|  | Proportion of recently released PLHIV who do not discontinue ART during post-release period | Calibration-based | Cohort studies, systematic review, modelling |
|  | Proportion of injections shared, non-incarcerated | Calibration-based | Modelling |
|  | Ratio of injections that are shared in prison vs non-incarcerated | Calibration-based | Assumed |
|  | Proportion of PWID never incarcerated | Calibration-based | Cross-sectional study, systematic review |
|  | Duration of incarceration and post-release period, by PWID status | Calibration-based | Surveillance data, cross-sectional study, cohort studies |
|  | Rate of reincarceration, by PWID status | Calibration-based | Cohort study, systematic review |
| ART=antiretroviral therapy, FSW=female sex workers, IBBA=integrated behavioural and biological assessment, IPV=intimate partner violence, IRR=incidence rate ratio, OAT=opioid agonist therapy, OR=odds ratio, PLHIV=people living with HIV, PWID=people who inject drugs, RCT=randomised controlled trial, RR=relative risk, STI=sexually transmitted infection, WHO=World Health Organization | | | |

**Table S6. Data on HIV epidemiology and structural determinants used to calibrate the models.**

| Reference | Calibration data related to HIV epidemiology | Calibration data related to structural determinants, interventions, and their effects |
| --- | --- | --- |
| a) Static approaches to representing structural determinants | | |
| Stover et al., 2021[55] | HIV prevalence, overall and by age from surveys, surveillance, and routine testing, probability of HIV transmission from systematic review and meta-analysis per act | None |
| Levy et al., 2021[51] | Adult population size, number of HIV infections, all-cause mortality, and percent treated from surveillance 2004-17 | None |
| Ronoh et al., 2020[58] | HIV prevalence among youth in Kenya 1990-2013 from surveillance | None |
| Vassall et al., 2014[56] | HIV and STI prevalence from IBBA survey data for FSW and clients (2005/6, 2007, 2008, 2011), and the FSW HIV prevalence ratio between Round 1 and later rounds | None |
| Wirtz et al., 2014[57] | HIV prevalence among FSW from surveillance 2007-2010 | None |
| Decker et al., 2013[49] | HIV prevalence among adults from surveillance 2008-2010 | None |
| Strathdee et al., 2010[5] | HIV prevalence among FSW in Ukraine 2004-2008 from cross-over experimental study and surveillance | None |
| b) Stratification-based approaches to representing structural determinants, where the modelled population could experience one level of exposure, with some movement between exposed and non-exposed states | | |
| Rigby and Johnson, 2017[52] | HIV prevalence by age in South Africa in 2012 | Proportion ever exposed to intimate partner violence and duration to onset of violence in relationships |
| Stone et al., 2022[53] | Calibrated to sampled values of HIV prevalence among PWID from a systematic review and meta-analysis | Calibrated to sampled values of the proportion of PWID unstably housed |
| c) Stratification-based approaches to representing structural determinants with multiple exposure histories | | |
| Shannon et al., 2015[4] | HIV prevalence in Canada among FSW (2010-12) and among PWID FSW (1997, 2004, 2006), in India among FSW (2005, 2008, 2010) and clients (2007), and in Kenya among FSW (1989, 1993-5, 1996-2000, 2005-6) and among FSW who binge drink (2005-6), proportion of PLHIV on ART | No, but the model was cross-validated using data on the prevalence of violence |
| Ward et al., 2022[54] | HIV prevalence among PWID, coverage of OAT and ART, PWID population size, in different years in each setting | OR for HIV prevalence between PWID ever or never incarcerated, proportion of community PWID ever incarcerated, overall and by injecting duration, and the number of times prisoners (Kyrgyzstan) or community PWID (other settings) have been incarcerated, in different years in each setting |
| Adams et al., 2021[44] | HIV prevalence among men and women in the US in 2012 and 2010 | None |
| Bernard et al., 2020[46] | HIV prevalence, awareness, and treatment, by PWID status in Washington | Rates of misdemeanor and felony arrests, number incarcerated in Washington |
| Adams et al., 2018[45] | HIV prevalence among men and women in the US in 2012 and 2010 | None |
| Borquez et al., 2018[48] | HIV prevalence among PWID (2005, 2006), HIV incidence among PWID (2014), proportion of new infections attributable to sexual transmission (2006) | Proportion of PWID ever incarcerated, by duration of injecting and sex, HIV prevalence among ever incarcerated PWID, relative HIV prevalence among ever vs never incarcerated PWID, from baseline cohort data in Mexico in 2011 |
| Altice et al., 2016[47] | ART coverage (2011, 2015) | HIV prevalence among PWID never and previously incarcerated PWID (2013) and currently incarcerated PWID (2011) |
| Dolan et al., 2016[50] | HIV prevalence among PWID | HIV prevalence among incarcerated PWID, HIV incidence in prison |
| ART=antiretroviral therapy, FSW=female sex workers, IBBA=integrated behavioural and biological assessment, OAT=opioid agonist therapy, OR=odds ratio, PWID=people who inject drugs, STI=sexually transmitted infection, | | |
