# Supplementary material for: From conceptualising to modelling structural determinants and interventions in HIV transmission dynamics models: a scoping review and methodological framework for evidence-based analyses
Source: BMC Med. 2024 Sep 19;22:404. doi: 10.1186/s12916-024-03580-z (PMC11414142; doi:10.1186/s12916-024-03580-z)
Supplement: Supplementary file 3 — Additional file 3: Figure S1. PRISMA-ScR checklist for the scoping review. [file 12916_2024_3580_MOESM3_ESM.docx]

**Additional file 3: Supplementary figures**

**Figure S1. PRISMA-ScR flowchart for the scoping review.** Screening identified 17 unique modelling studies that used 13 different models to estimate the impact of structural determinants or interventions, including criminalisation and incarceration, stigma and discrimination, gender-based violence, homelessness, and education and empowerment.

Articles identified (n = 2510) from:

Embase (n = 1891)

Medline (n = 619)

Duplicate records removed before screening

(n = 401)

Articles screened

(n = 2109)

Non-relevant records excluded by title and abstract

(n = 2031)

Full texts assessed for eligibility

(n = 78)

Records excluded (n = 60):

Structural determinants not modelled (n = 37)

Only modelled scale-up of biomedical interventions (e.g., PrEP, ART, OAT) (n = 5)

Not a transmission dynamic model (n = 4)

Modelled incarceration but did not estimate its impact (n = 3)

Did not model HIV (n = 3)

Not a modelling study (e.g., protocol, framework) (n = 3)

Abstract only (n = 3)

Not peer-reviewed (e.g., preprints) (n = 2)

Not in English (n = 1)

Unique studies included in review (n = 17)

Unique models included in review (n = 13)

**Identification**

**Screening**

**Included**
